# New Strategies for the Treatment of Atrial Fibrillation

**DOI:** 10.3390/ph14090926

**Published:** 2021-09-15

**Authors:** Norbert Jost, Torsten Christ, János Magyar

**Affiliations:** 1 Department of Pharmacology and Pharmacotherapy, Faculty of Medicine, University of Szeged, 6725 Szeged, Hungary; 2 Department of Pharmacology and Pharmacotherapy, Interdisciplinary Excellence Centre, University of Szeged, 6725 Szeged, Hungary; 3 ELKH-SZTE Research Group for Cardiovascular Pharmacology, Eötvös Loránd Research Network, 6725 Szeged, Hungary; 4 Institute of Experimental Pharmacology and Toxicology, University Medical Center Hamburg-Eppendorf, 20246 Hamburg, Germany; t.christ@uke.de; 5 DZHK (German Center for Cardiovascular Research), Partner Site Hamburg/Kiel/Lübeck, 20246 Hamburg, Germany; 6 Department of Physiology, Faculty of Medicine, University of Debrecen, 4032 Debrecen, Hungary; magyar.janos@med.unideb.hu; 7 Department of Sport Physiology, Faculty of Medicine, University of Debrecen, 4032 Debrecen, Hungary

**Keywords:** atrial fibrillation, atrial electrical remodelling, atrial fibrosis, antifibrillatory drugs, investigational drugs

## Abstract

Atrial fibrillation (AF) is the most prevalent cardiac arrhythmia in the clinical practice. It significantly contributes to the morbidity and mortality of the elderly population. Over the past 25–30 years intense effort in basic research has advanced the understanding of the relationship between the pathophysiology of AF and atrial remodelling. Nowadays it is clear that the various forms of atrial remodelling (electrical, contractile and structural) play crucial role in initiating and maintaining the persistent and permanent types of AF. Unlike in ventricular fibrillation, in AF rapid ectopic firing originating from pulmonary veins and re-entry mechanism may induce and maintain (due to atrial remodelling) this complex cardiac arrhythmia. The present review presents and discusses in detail the latest knowledge on the role of remodelling in AF. Special attention is paid to novel concepts and pharmacological targets presumably relevant to the drug treatment of atrial fibrillation.

## 1. Introduction

Atrial fibrillation (AF) is a cardiovascular disease, with relatively high incidence (approximately 1.5–2.2%) in the whole population [1]. It is expected to be increased in the near future [2,3]. In principle, AF is not a lethal disease but is far from being considered as a benign illness, since it is major contributor to the elevated risk of stroke due to thromboembolism and of congestive heart failure [4,5]. AF is connected with the highest rate of hospitalization, which is increased especially at advanced age [4,6].

**Figure 1:**
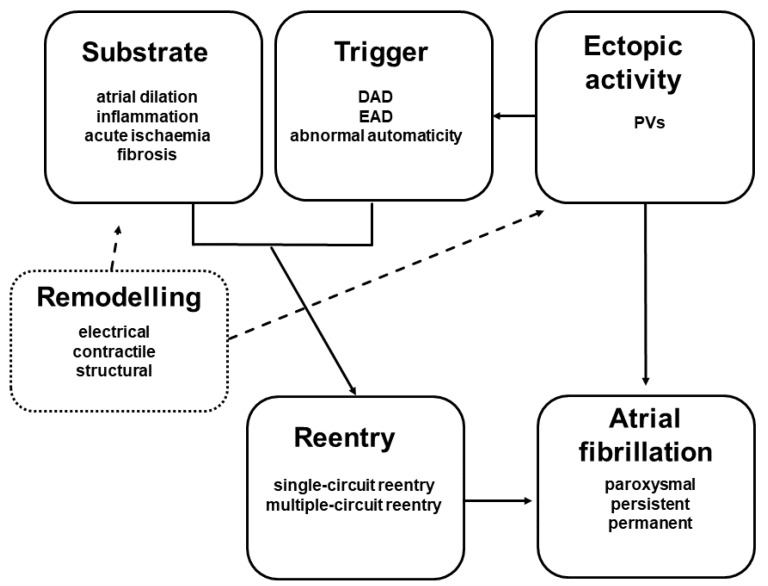
General scheme presenting the main factors, which are implicated in provoking and sustaining atrial fibrillation (AF). AF is usually initiated by spontaneous ectopic beats (extrasystole) originating from a pacemaker region (PVs, left upper pulmonary vein) and later is maintained by mechanism of atrial remodelling. The atrial fibrillation forms (lone, persistent and permanent) are promoted by single/mother wave and/or multiple wave/circuit re-entries. The main factors that may promote reentry are atrial ischaemia and inflammation, while the structural/morphological remodellings are sustained by atrial (micro)fibrosis and left atrial dilation. DAD and EAD—delayed and early afterdepolarization; PVs—pulmonary veins.

AF occurs when an atrial ectopic beat interferes with an anatomic and/or functional obstruction, and as a consequence will trigger the re-entry of the impulse/excitation wavefront (Figure 1). The basic mechanisms that underlie AF are as follows: (a) increased automaticity; (b) triggered automaticity, which are early (EAD) and delayed (DAD) afterdepolarizations.

Several studies revealed that pulmonary sleeve veins contain cardiomyocytes with increased pacemaker activity, which thereby behave as ectopic foci and may induce single or multiple re-entry circuits [7,8]. Nowadays there are two main theories, which may explain the persistence of AF. The first is the so-called “leading circle” theory conceived by Allessie, which is based on the existing single and multiple re-entry circuits [7]. The second theory elaborated later by Jalife and was formulated as cardiac rotor theory [9]. Persistent or permanent atrial fibrillation is maintained by the phenomenon entitled atrial remodelling comprising the following three main elements: electrical, contractile and structural remodelling. These alterations initiated by atrial remodelling play a major role in the self-perpetuation/renewal of the atrial arrhythmia and resistance to sinus rhythm conversion. The main elements that contribute to AF remodelling consist of: (a) the functional and structural changes and injuries of cardiac atrial cells (sarcolemmal ion channels, signalling and functioning proteins), cell-surface coupling structures (gap-junctions), the extracellular matrix and the endocardial endothelium; (b) the dysfunction of neurohumoral system, from which mainly the renin-angiotensin-aldosterone system (RAAS) is affected.

The first part of the present review briefly discusses novel aspects in the pathophysiology of atrial remodelling with particulars of electrical alterations and atrial fibrosis. The second part summarizes the currently available therapeutic options and strategies for developing novel pharmacologic agents suitable to prevent and/treat atrial remodelling.

## 2. Mechanisms of Atrial Remodelling

### 2.1. Electrical Remodelling

Briefly, we may define the term of the remodelling in AF as the main factor responsible for the progressive nature of the arrhythmia. Several studies described the most important aspects of the phenomenon of AF remodelling, which were shown comprising three major components—electrical, contractile and structural remodelling—that synergistically contribute to the generation of the vulnerable substrate [10,11].

**Figure 2:**
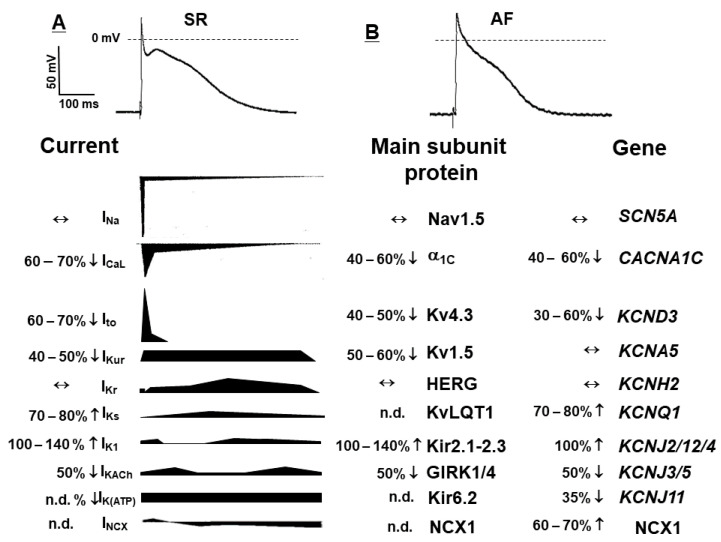
The most important transmembrane ionic inward and outward currents determining atrial action potential in sinus rhythm (SR, **A**) and in permanent atrial fibrillation (AF, **B**). The ion channel/current (functional elements) density changes are presented in bottom left column, while the expression level current subunit forming proteins and mRNA genes, respectively, are depicted in middle and right columns. The corresponding pictograms near each ion current name present the magnitude and time course of the current reflection of relative real sizes. **↓**—decreased current or downregulated protein/gene, respectively; **↑**—increased current or upregulated protein/gene, respectively; **↔**—unchanged current, protein or gene, respectively; n.d.—no data.

*Electrical remodelling* is caused by AF induced changes of several inward and outward ion channels with the subsequent abbreviation of the action potential (AP) and more negative RMP [5]. The most important changes, which should be noted are the alterations due to the downregulation of the plateau currents and upregulation of several repolarizing currents (Figure 2). Accordingly, there are three major changes that underlie the AP shortening and triangularization: (a) downregulation of the L-type inward calcium current (I_Ca,L_); (b) upregulation of the inward rectifier potassium current (I_K1_); (c) activation of the constitutively active I_K,Ach_ (reviewed in detail by [10,12,13,14]).

Several reports indicate that electrical remodelling (manifested in shortened atrial action potential/refractoriness) is usual fully reversible after the conversion to sinus rhythm (SR); however, when AF becomes more stable the calcium overload (resulted from altered calcium homeostasis) will be the main source responsible for initiating arrhythmogenic re-entry circuits [14]. Indeed, high Ca^2+^ concentration will bind calmodulin, which subsequently will activate the calcineurin and trigger the signalling events responsible for perpetuation of the AP shortening and the AP hyperpolarization. The latter is the result of the increase in the inward rectifier potassium current (I_K1_) [14].

However, there were some studies, which challenged the major role of electrical remodelling in initiating AF. For example, Healey et al. [15] reported that they did not find major differences in electrophysiological parameters between patients after two years AF and those without intermittent atrial fibrillation.

When considering the effect of ion channel alteration on atrial electrical remodelling we must emphasize that there are many diseases induced by ion-channel genetic mutations as Brugada syndrome (SCN5 gene mutation), Andersen–Tawil syndrome (KCNJ2 gene mutation), catecholaminergic polymorphic ventricular tachycardia (CVPT, RYR2 gene mutation), etc. [16,17]. The genetic predisposition for AF has not been appreciated for a long time, nevertheless many papers reported that rarely, AF can develop in some families at a relatively young age despite the absence of any evidence of structural heart disease or any other apparent etiology [18]. However, nowadays there is much evidence, which undoubtedly demonstrate that ion-channel gene mutations may also be primary or contributing factors for inducing and/or maintaining AF. There are more and more familial genetic investigations, which strengthen that ion channel mutations are the cause and/or promotors for AF susceptibility. The genetic predisposition of AF can be caused by a single clinical aspect (monogenic disease when AF is the primary disease of one or more family members) or can be as a result of another cardiac genetic disease (multiple rare genetical disease).

In 2003, a prospective study was performed involving more than 5000 individuals whose parents were enrolled in the Framingham Heart Study [19]. The results confirmed having a parent with AF approximately doubled the four-year risk of developing AF even after adjustment for risk factors such as hypertension, diabetes mellitus, or myocardial infarction [19]. Another recent multicenter prospective observational cohort study including 1722 nonvalvular AF patients from February 2008 to August 2019 in Italy, showed that family history of AF is evident in more than 20% of patients and was associated with an increased risk for cardiovascular events and mortality [20]. The observation that AF etiology may have also genetic origins is important since there are great promises in genetic therapies, which can be adapted also in the treatment of genetical mutation induced forms of AF [21].

### 2.2. Contractile Remodelling

Calcium overload is also the main factor of atrial cardiomyocytes in the initiation of the Ca^2+^-sparks. The phenomenon is induced by increased activation of the ryanodine receptors in the sarcoplasmic reticulum. These Ca^2+^-sparks most probably contribute to the contractile remodelling observed in AF [22]. We must emphasize that in heart failure—a disease known to influence AF—the altered level in intracellular Ca^2+^ handling before the onset of AF is different from that observed after the onset of AF [23]. Furthermore, another study revealed that a completely different dynamics for intracellular Ca^2+^-handling is characteristic during AF progression or after its termination [24]. Consequently, one can distinguish the following three steps: (a) Ca^2+^-overload; (b) remodelling; (c) steady state. In the steady the recovery of calcium contraction also happens in three phases: (a) calcium unloading; (b) reverse remodelling; (c) full recovery [24]. These observations are in good agreement with those found in human atrial tissue [25]. These reports may serve basis for new medication therapeutic options [26,27].

Another direct sequel of the AF-induced altered calcium handling, referred as contractile remodelling, includes loss of atrial contractility, which will result in a subsequent increase in compliance and, in the end atrial dilation. In a six-week atrial tachycardia model, the fast downregulation of L-type Ca^2+^ channels was reported. The downregulation of the channels was associated, with substantial alteration in Ca^2+^-transients and especially in cell contractility [28,29]. Since I_CaL_ is the main element for Ca^2+^-release from the SR (Ca^2+^-induced Ca^2+^-release, CICR), it is concluded that the AF induced reduction of I_CaL_ should be the main pathogenetic factor for AF-induced contractile remodelling. Accordingly, Schotten et al. came to the conclusion that the loss of atrial myocardial contractility is a result of the I_CaL_ reduction and impaired Ca^2+^-homeostasis, and consequently the reduced contractility leads to atrial dilation, thereby “electrical and contractile remodelling, which go hand in hand” [30].

### 2.3. Structural Remodelling

Chronic atrial stretch and anatomical deformation are the main factors that cause the signalling pathways to determine the cellular hypertrophy and interstitial, intercellular fibrosis, jointly referred as structural remodelling [31]. The structural remodelling is a principal factor for the progressive nature (paroxysmal/lone **→** persistent **→** permanent) of AF. It should be noted that structural remodelling is the cachet of heart failure and of other chronic heart diseases, which means that the occurrence of AF is significantly supported in these diseases.

Tissue fibrosis represents the common endpoint in different heart diseases as, for example, in heart failure from hypertensive, coronary and diabetic patients. In the majority (at least two-thirds of cases), AF is associated to a pre-existent organic heart disease that promotes the development of the vulnerable basis for AF, whereas lone/paroxysmal AF occurs in one-third of patients. There is a good deal of experimental and clinical evidence that highlights the association between atrial fibrosis and AF [30,32,33,34].

The AF-related atrial fibrosis is the result of a complex interaction between many largely known profibrotic factors and/or signalling pathways as inflammation or oxidative stress. For example, it was reported that both atrial and ventricular fibrosis may be induced by: (a) renin angiotensin aldosterone (RAAS) system (angiotensin II acting on AT-1 receptors and aldosterone acting on the mineralocorticoid receptor); (b) TGF-beta1 (transforming growth factor, TGF) known to stimulate collagen production via SMAD (Mothers Against Decapentaplegic is a protein from the SMAD family) pathway. In addition, AF has been shown to be associated with high levels of inflammatory serum biomarkers, thus many anti-inflammatory agents exert an antifibrotic effect [35]. However, we must emphasize that the non-steroidal anti-inflammatory drugs (NSAIDs) were shown to increase, in a large population-based case-control study, the elevation of the relative risk of AF or atrial flutter (AFlu) [36].

Recent intensive studies revealed the role of miRNA in AF-associated electrical and structural remodelling. MiRNAs are a class of small, non-coding RNA (miRNA) molecules that silence gene expression at the post-transcriptional level [37]. It was reported that the downregulation of miRNA-26 was associated with increased density of inward rectifier potassium current (I_K1_). The upregulation of miR-328 elicited inward L-type calcium current (I_Ca,L_) diminution in both human and animal atrial samples [38]. Shan et al. [39] reported a profibrotic response in a dog model of AF induced by rapid pacing and nicotine administration. The AF-induced fibrosis was associated with significant upregulation of the expression level of TGF-beta1 and TGF-betaRII and substantial reduction (at least 60–70%) of the anti-fibrotic miR-133 and miRNA-590 levels.

In another study, it was reported that after eight weeks of experimental myocardial infarction in rats, the knockdown of atrial miRNA-21 suppresses atrial fibrosis and the duration of AF [37]. Another study reported that in atrial myocardium of 4 subjects with permanent AF the expression level of miRNA-499 to be significantly upregulated, while the expression of the small-conductance calcium-activated potassium channel 3 (SK3) proteins to be downregulated [40]. However, it is still not clear whether this finding might contribute to the electrical remodelling in AF. Accordingly, it has been also observed that miRNA-29 was substantially downregulated in patients with chronic heart failure and atrial fibrillation; moreover, the decreased miRNA-29b expression level in canine atrial fibroblasts induced an upregulation in the collagen expression level, thereby contributing to atrial fibrotic remodelling. Therefore, the authors concluded in this study that miRNA-29 likely plays a role in atrial fibrotic remodelling and may prove valuable as a potential biomarker and/or therapeutic target in the future [2].

### 2.4. The Connection between Electrical, Contractile, and Structural Remodelling

**Figure 3:**
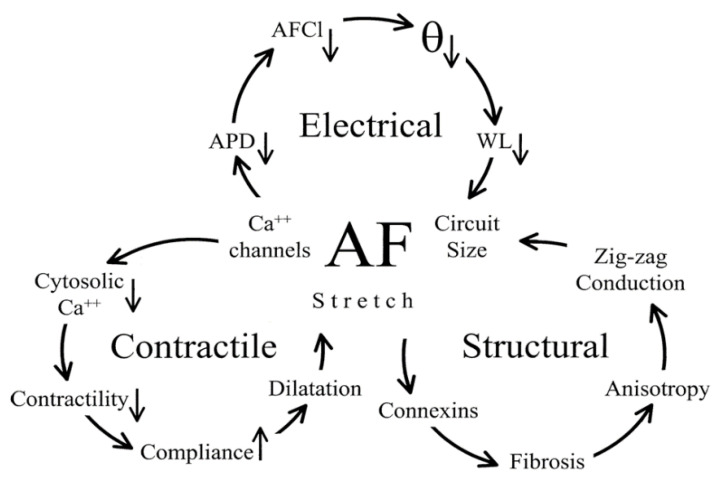
Three proposed positive feedback loops to present the progredient development of atrial remodelling in AF. The electric and contractile remodelling are associated with the downregulation of L-type Ca^2+^-channels which as a result will cause the alteration of the whole Ca^2+^-homeostasis process. The loss of contractility and increase in compliance will induce the stretch of the atrial myocardium, which is considered to be the main inducing factor of the structural remodelling. The resulting intra-atrial circuits due to a reduction in wavelength and increased non-uniform tissue anisotropy and even zig-zag conduction (anatomical basis of re-entry or rotors). Reprinted with permission from ref [10]. Copyright year 2002, Oxford Academic.

It was reported that electrical and contractile remodelling have two phases [31]. The first is the rapid electrical and contractile remodelling, which occur in a short time (within minutes to hours) after the first AF episodes. The second phase electrical and contractile remodelling develops in a much longer time scale (from days to weeks). There are several clinical studies, which have reported that fortunately the electrical and contractile remodelling usually become fully reversible after conversion to SR (electrical and/or pharmacological) in a relatively long time frame [30,32,33,34]. Since structural remodelling is the result of a complex process, it develops much slower, usually within a minimum of 3–5 months. Unfortunately, these structural/morphological alterations (especially microfibrosis and left atrial dilation) of the atrial tissue are predominantly irreversible, therefore the structural remodelling goes with the tough recurrent, especially permanent forms of AF. Figure 3 presents the cascade relation between electrical, contractile and structural remodelling [10].

## 3. Therapeutic Options to Prevent and Stop Atrial Remodelling

### 3.1. Back to Sinus Rhythm or Just Control Ventricular Rate

Rhythm control is a therapeutic option and an intervention to stop atrial fibrillation, i.e., to re-establish the normal sinus rhythm (SR). Rate control aims to slow down conduction in the AV-node to reach physiological ventricular rate. Slowing of AV node conduction can be achieved by application of various types and classes of antiarrhythmic drugs. The drugs used most frequently are β-blockers and some Ca^2+^-channel blockers, like verapamil [41]. It is now accepted that ectopic triggers originating from pulmonary veins can cause atrial extrasystoles and consequently trigger AF. Eliminating the triggers coming from such ectopic foci can terminate AF and, hence, provide rhythm control. Several classical antiarrhythmic drugs which include especially the Na^+^ channel blockers or the so-called multiple ion channel blockers (for example amiodarone) can be used to reach this goal. The commonly accepted wavelet concept [7] predict that short effective refractory period and/or slowed conduction will favour the likelihood of generating re-entries.

### 3.2. New Approved Drugs and Investigational Compounds to Convert AF

All existing antiarrhythmic agents used for the treatment of AF are far from being ideal, since they show not only a very limited efficacy to stop AF, but they possess a relevant safety issue. In general, all antiarrhythmic drugs effective in terminating ventricular arrhythmias may also successfully suppress AF by lengthening atrial effective refractory period (AERP) and by slowing atrial tissue conduction. However, to minimize ventricular proarrhythmic effects, an atrial selective action would be needed. Such a characteristic was referred as the “atrial selective drug concept”, which means that these drugs are expected to block ion currents existing in the atria but not in the ventricles (or they are at least less relevant in ventricles). In addition, drugs used for rhythm control should lack other cardiac side effects like negative inotropy or extra cardiac organ toxicity. Importantly, atrial selective drugs to stop AF should be tolerable in patients having common cardiac comorbidities such as coronary artery disease or heart failure.

**Figure 4:**
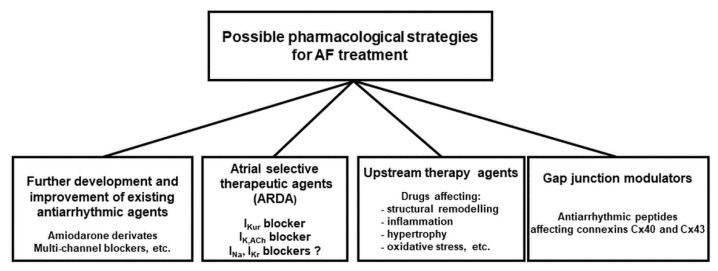
Possible pharmacological investigational strategies for developing new antiarrhythmic drugs for treating AF.

There are several new promising concepts to design new antiarrhythmic drugs to stop AF more efficiently but with fewer side effects. As Figure 4 summarizes, this goal can be reached by: (a) further developing and improving existing antiarrhythmic agents (specific or multiple ion channel blockers); (b) new compounds which act in an atrial-selective manner (atrial repolarizing delayed agents—ARDA); (c) compounds that mediate their action not via non-ion channel, but target regulating upstream processes (inhibiting inflammation or fibrosis); (d) new compounds that act not via classic ion channels but rather via gap-junctions (the latter approach being considered as the most recent attempt to treat AF pharmacologically).

We will briefly comment on the principles, mentioned above. Table 1, tries to sum up some key characteristics of the drugs, addressed.

#### 3.2.1. Blockers of Specific Ion Channels vs. Multiple Ion Channel Blocker to Stop AF

The principal action for these drugs is to prolong repolarization (i.e., Class III compounds). During last two decades, there were several novel compounds or investigational drugs developed. However, most of these compounds have been abandoned because all of them bear an unacceptable risk for induction of ventricular proarrhythmic events, especially *Torsades de Pointes*-type tachyarrhythmias.

##### Selective I_Ks_ Blockers

**Figure 5:**
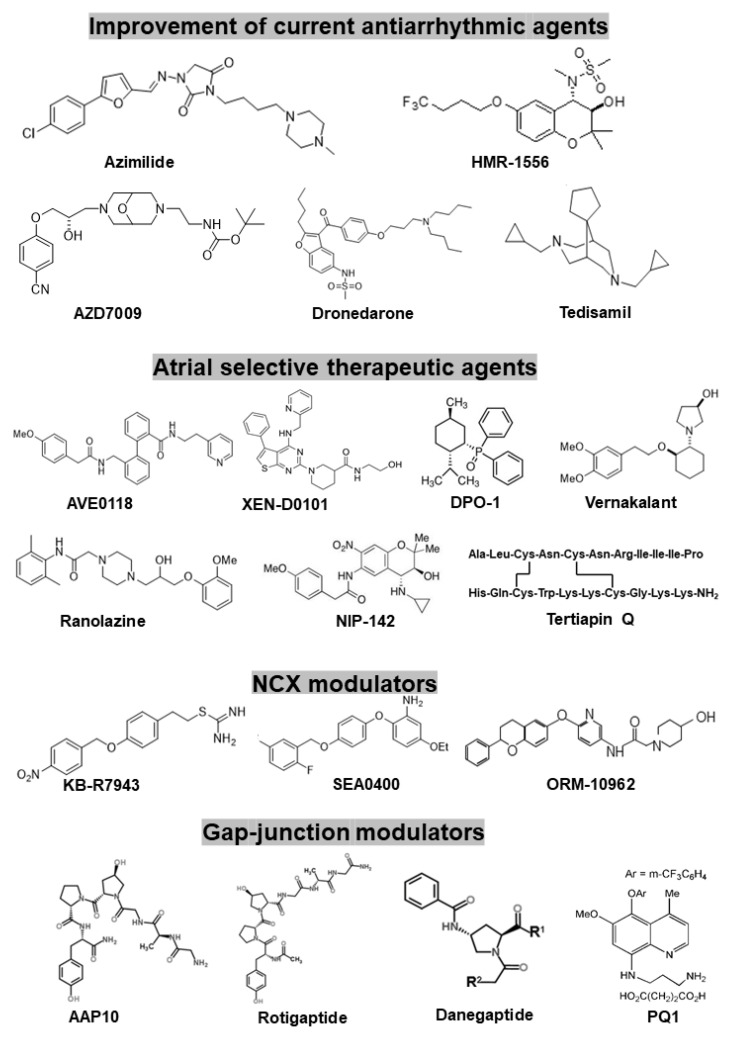
The chemical structures of the reviewed drugs in the present paper (specific-, multiple-, atrial selective ion channel blocker drugs and gap-junction modulator antiarrhythmic peptides.

**Azimilide** (Procter & Gamble, Cincinnati, OH, USA, specific I_Kr_ and I_Ks_ blocker, Figure 5). The development of this drug was inspired by the elegant observation that blockers of I_Kr_ show larger prolongation of refractoriness at low rate (so-called *Sanguinetti’s* hypothesis, [42]), limiting their effectiveness in AF. In contrast, it was presumed that the block of I_Ks_ would not show such a reverse rate-dependent action. However, it became evident that azimilide blocks not only I_Ks_ but I_Kr_ as well. Later reports demonstrated that azimilide, like amiodarone, blocks also calcium channels and more importantly sodium channels in a use-dependent manner [43,44]. Results from the first somewhat encouraging studies in patients with AF [45,46], could not be confirmed by several follow-up studies (e.g., the ALIVE trial). Now it seems clear that azimilide will never be a useful compound to stop AF [47,48,49].

**HMR-1556** (Hoechst-Marion-Rousell, Kansas City, MO, USA, I_Ks_ blocker, Figure 5). Contrary to azimilide HMR-1556 is a highly selective blocker of I_Ks_. The idea of selective block of I_Ks_ attracted much attention by pharmaceutical companies. In fact, HMR1556, the first powerful and selective blocker of I_Ks_ was used in many preclinical studies. Indeed, numerous patch-clamp investigations demonstrated that HMR-1556 effectively blocks I_Ks_. Only at concentrations much higher needed to block I_Ks,_ the compound also inhibits I_to_ and to some extent the sustained outward current I_sus_, and I_CaL_ currents [50]. HMR-1556 prolonged the atrial effective refractory period (AERP) in dog atria. In a canine model of vagal AF HMR-1556 exerted a modest effect on the duration of induced AF. However, the compound was only effective in the presence of intact β-adrenergic stimulation [51].

**AZD7009** (Astra Zeneca, Cambridge, UK, I_Kr_ and I_Na_ blocker, Figure 5). The main pharmacological effect of AZD7009 is to block I_Kr_ and also I_Na_ (classic positive rate-dependent I_Na_ block). The compound is effective at micromolar concentrations. Later studies revealed that the compound also blocks several repolarizing currents such as I_to_, I_Kur_ and I_Ks_ in higher concentrations [52,53]. As expected from such a profile, AZD7009 concentration dependently reduced V_max_ and increased APD in atria (dog) and the APD prolongation by AZD7009 did not show positive frequency dependence [54]. First clinical trials demonstrated AZD7009 to be able to convert persistent atrial fibrillation in humans [55,56,57]. It is unclear whether the latter effect results solely from activity on Na^+^ channels.

#### 3.2.2. Amiodarone-Like Multichannel Blockers

The undoubted success of the multichannel blocker amiodarone stimulated effort to follow the idea that a simultaneous blockade of several specific inward and outward currents may result in a more effective electrophysiological profile compared to single channel blockers [58].

**Dronedarone** (Sanofi Aventis, Paris, France, Figure 5) has so far been the most advanced compound. The development has been based on the idea to get a new multichannel blocker as effective as amiodarone, but devoid of its considerable extra cardiac toxicity.

The starting point for designing dronedarone was to keep the molecule of amiodarone but without containing an iodine, since it is generally accepted that the iodine molecule of amiodarone is responsible for many of the severe extra-cardiac (thyroid, ocular, pulmonary, and hepatic) toxicities of the drug [59]. Electrophysiological studies in vitro showed that dronedarone possesses quite similar acute and chronic effects as reported earlier for amiodarone [60,61]. In dog ventricular preparations, dronedarone indeed acts as a multichannel blocker. Dronedarone blocks I_Ca,L_ and I_Kr_, and reduces the maximum upstroke velocity in a frequency-dependent manner, indicating block of I_Na_ [61]. Based on these promising preclinical investigations and the lack of iodine-associated toxicity dronedarone has been recommended for combating atrial arrhythmias. ADONIS and EURIDIS, two large multicentric trials, reported the superior efficacy of dronedarone to prevent the reoccurrence of AF. The drug did not affect significantly the QT interval, therefore its Class III type proarrhythmic side effect (torsadogenic potency) was very low. [62,63]. Moreover, the next trial (ATHENA) reported prolonged time (24% compared to placebo) to the first cardiac hospitalization or cardiac death [64]. However, we must emphasise that this observation was somewhat surprising, since other groups reported that the initial congener (“Mother”) compound amiodarone remains net superior to dronedarone [65,66].

As a result of ATHENA trial dronedarone obtained FDA approval in the USA. The main indication was prevention of hospitalizations due to recurrent AF (but not to convert AF), therefore, dronedarone was never allowed to be marketed in USA as primary anti-AF agent [66]. Later, some severe non cardiac side effects were associated with dronedarone administration, from which we must stress reported cases of severe liver toxicity making immediate liver transplantation necessary [67]. However, recent real-life data indicate that dronedarone liver toxicity is not different from that of other antiarrhythmics like amiodarone [68].

**Tedisamil** (Figure 5) was initially developed as anti-ischemic and bradycardic drug (by Solvay Pharma-Kali Chemie AG, Hannover, Germany). Originally, it was presumed to be only a selective I_to_ current blocker, which would be beneficial, against AF, since I_to_ currents are more abundantly expressed in the atrium than in the ventricle. However, additional studies reported that tedisamil possesses multichannel, especially complex potassium (I_to_, I_Kur_, I_Kr_, I_Ks_ and even I_KATP_) blocking properties, and also blocked fast sodium channel with slow offset kinetics and with onset kinetics intermediate between that of slow (quinidine) and fast (lidocaine) antiarrhythmic drugs, corresponding to Class I A/B antiarrhythmic action [69]. In conclusion, tedisamil possesses strong Class III antiarrhythmic (antifibrillatory) effect associated with increased APD, QT interval and refractoriness, but the additional sodium channel block can also decrease the dispersion of repolarization and incidence of early afterdepolarizations, which may counterbalance the possible inherent ‘torsadogenic’ potential of the compound due to its I_Kr_ blocking property [69,70,71,72]. Probably as a result of this “amiodarone-like” multichannel blocking properties, tedisamil did not show a reverse use-dependence of APD prolongation. Based on several dog studies [73,74] and human clinical phases 2 and 3 trials [75], in 2006 Solvay Pharma initiated unsuccessfully the FDA approval [76] for tedisamil as potential antiarrhythmic drug for treating atrial flutter and atrial fibrillation with Pulzium^TM^ trademark name [77]. According to FDA review, the main concerns against tedisamil medication were: “The pooled safety data for tedisamil showed a disproportionate increase in adverse events among women, including elevated rates of Torsades de Pointes, hypotension, tachycardia and bradycardia.” [77]. Therefore, we may conclude that tedisamil seems unlikely to be a beneficial new drug combating AF.

#### 3.2.3. Can Atrial Selective Ion Channel Block Stop AF?

The most promising strategy to treat AF that avoids ventricular proarrhythmic side effects is the development of drugs known as “atrial selective drugs”. This concept exploits distinct differences in expression patterns of individual ion channels and their different contribution to refractoriness between atrial and ventricular myocytes.

Accordingly, the aim of atrial specific ion channel blockers can be reached by designing compounds that modify (block or activate) function of ion currents selectively contributing to repolarization only in human atrial cardiomyocytes, i.e., which are not or only weakly present in ventricle. Such atrial specific targets, which would be suitable for developing novel AF combating/preventing treatment include the following three known atrial specific ion currents: (a) the ultra-rapid delayed rectified potassium current (I_Kur_), (b) the acetylcholine-sensitive inward rectifier potassium current (I_K,ACh_), (c) the constitutively active I_K,ACh_ currents (i.e., which are active even in the absence of agonists at muscarinic receptors).

In keeping with literature data, there is no doubt that the channels responsible for I_Kur_ and I_K,ACh_ are exclusively or near exclusively present in atria, they are known to be absent in human ventricles. Therefore, they should be appropriate pharmacological targets for atrial selective ion channel blocker drugs (ARDA—atrial repolarization delaying agent).

In addition, there are other ion channels that are present both in atria and ventricles, but due to their differences in biophysics (kinetic properties, voltage dependence of activation and/or inactivation) or selective modulation of channels, some appropriate compound may evoke larger effects in atria than in ventricles. Consequently, such ion channel modulators may also fulfil the criteria of atrial selective antiarrhythmic drugs.

##### I_Kur_ Blockers

The ultra-rapid component of the delayed rectifier potassium current (I_Kur_) current was firstly, described by Wang and co-workers [78] and now is considered to be the most typical atrial selective transmembrane current [79]. Accordingly, many pharmaceutical companies have invested significant efforts in developing selective I_Kur_ blockers as novel pharmacological agents against AF. As a result, from the beginning of this century, many new selective I_Kur_ blocker compounds were developed and tested. The most investigated drugs or investigational compounds were: AVE0118, XEN-D101, DPO-1, vernakalant, ISQ-1, DPO-1, AZD7009, NIP-141, NIP-142, acacetin, etc.). However, we must emphasize that early reports, such as the one by Wettwer [80] questioned the effective APD lengthening property of I_Kur_ blockers.

**AVE0118** (Figure 5) is a biphenyl derivative compound, which was developed by Sanofi-Aventis. The AVE0118 blocks I_Kur_ at micromolar concentrations in both native human atrial cells and in Kv1.5 channel transgenic systems as well. In addition to the block of I_Kur_, the drug at similar concentration range also blocked I_to_ and I_K,ACh_ currents [81,82]. In several large animal models of permanent AF (dogs and goats), AVE0118 was reported to possess rate dependent atrial ERP lengthening and AF converting to SR potency with only minimal effect on ventricular refractoriness and QT interval. In addition, no AVE0118-related proarrhythmic effect was reported [83,84,85]. AVE0118 shortened APD and ERP in atrial tissue originating from patients in SR, while APD/ERP was only slightly prolonged in tissues from patients in AF [82]. This observation is in good agreement with a previous study performed with the non-selective I_Kur_ blocker 4-aminopyridine [80]. There are no clinical studies published with AVE0118 and it seems that its development as a possible antiarrhythmic drug has likely ceased. However, the compound was recently suggested as a new pharmacological tool to treat obstructive sleep apnoea [86].

**XEN-D0101** (Figure 5) and XEN-D0103 (chemical structure not disclosed) are two experimental compounds that were developed by a small R&D company (Xention Ltd., Cambridge, UK). It seems that these two molecules are the only highly selective Kv1.5 channel blocker agents so far. Accordingly, in several investigations it was clearly demonstrated that XEN-D0101 and XEN-D0103 selectively blocked I_Kur_ channels and prolonged the atrial ERP and decreased the duration of AF in dogs [87,88,89,90]. A first clinical study with XEN-D01013 in patients with paroxysmal AF have failed to reduce the burden of AF ([91]).

**DPO-1** (Diphenylphosphine oxide, Figure 5). DPO-1 blocks I_Kur_ rate-dependently at nanomolar concentrations in isolated human atrial myocytes. At higher micromolar concentrations, DPO-1 blocks other currents as I_to_. In human atrial tissue and not in the ventricle, DPO-1 induced plateau elevation and shortening in SR, and prolongation of APD in AF. In vivo investigations demonstrated that the compound successfully prevented and suppressed AF induced in a non-human primate model and atrial flutter in a dog AF model [92,93].

**Vernakalant** (RSD1235, Cardiome and Astellas, Deerfield, IL, USA, Figure 5) is the drug in the most advanced phase of investigation. It has been approved by the European authorities but not by the FDA for intravenous conversion of AF (for discussion see [94]). Vernakalant blocked Kv1.5 in a positive frequency-dependent manner [95,96]. However, its effects on transient potassium outward current in human atrial cardiomyocytes are rather small. Vernakalant clearly suppresses upstroke velocity in human atrial preparations, indicating relevant block of I_Na_ [95,96], so it may be regarded as a multichannel blocker rather than a selective I_Kur_ (ARDA type) blocker. Vernakalant possesses fast offset kinetics at sodium channels, thereby it was not expected to cause conduction disturbances and proarrhythmia at low heart rates [97,98]. However, vernakalant slowed conduction velocity at physiological heart rate in both atrium and ventricles of human hearts questioning atrial selectivity of the action of the drug [99]. Several clinical studies revealed the efficacy and safety of the vernakalant in conversion of AF. The AVRO study (phase III clinical study) demonstrated that vernakalant when compared to amiodarone, possesses superior efficacy for acute conversion of recent-onset AF [100,101].

In another small study, it was demonstrated that vernakalant with combination of electrical cardioversion is safe and efficacious [102], therefore vernakalant was approved for clinical practice in the European Union in 2010, but not in the United States of America [103].

##### Sodium Channel Blockers

The “modulated receptor hypothesis” revealed that fast sodium channel blocker drugs (like Class IA antiarrhythmics) bound preferable to open (activated) or closed (inactivated) states of the I_Na_ channel. Since I_Na_ channels are present in atrial and ventricular myocytes as well, the atrial selectivity of I_Na_ channel blockers is not a self-explaining concept. Based on the hypotheses, formulated by Antzelevitch and co-workers [104], we must consider the following two mechanisms potentially lead to atrial selective Na^+^-channel block: (a) human atrial tissue has a slightly less negative (depolarized) resting potential than ventricular tissue [105]; (b) potential for half-maximum inactivation of I_Na_ is more negative in atrial vs. ventricular cardiomyocytes.

Taking these factors into consideration, we may say that atrial tissue will recover faster from inactivation (due to its less negative membrane potential), therefore I_Na_ blockers—which binds preferably to inactivated channel state—will exhibit a larger blocking effect in atria than in ventricles [104]. For additional discussion, also compare Ravens and Christ [106].

**Ranolazine** (Figure 5) is a compound that initially was developed as an antianginal drug. Soon it was reported that ranolazine reduced transmural dispersion of APD and successfully suppressed ventricular EAD triggered arrhythmias [107]. Several patch clamp studies revealed that ranolazine blocks primarily the late I_Na_ (I_Na,L_) but there is no doubt that the compound blocks also several other currents as I_Kr_, I_Ks_, and even possibly L-type calcium current (I_CaL_) [108]. Furthermore, ranolazine at concentrations frequently used to block I_NaL_ is an effective β-adrenoceptor antagonist [109]. Ranolazine was also shown to reduce intracellular sodium and calcium overload during ischemia and effectively suppressed triggered activities, such as EADs. These properties were associated with block of the late I_Na_ current by the compound. However, from the complex pharmacology mentioned above, interpretation is complicated. In human AF, I_NaL_ was found to be larger [110]. The exact extent of AF-associated increase in I_NaL_ is a matter of debate [111]. More importantly, contribution of I_NaL_ to refractoriness is hard to judge since a selective blocker of I_NaL_ is still missing. Several studies performed in isolated single and multicellular wedge perfusion canine ventricular preparations also reported that ranolazine reduced the proarrhythmic transmural dispersion of AP repolarization [104]. More importantly in the context of AF, experiments in canine isolated perfused atrial and ventricular preparations have suggested that ranolazine shows stronger effects on atrial than on ventricular sodium channels [112]. Clinical studies showed only moderate efficacy of ranolazine in counteracting AF [113]. Nevertheless, ranolazine was shown to possess AF-activity both alone [114] or in combination with other drugs such as amiodarone and dronedarone (HARMONY Trial, [115]).

We must not forget that vernakalant (RSD1235, Cardiome and Astellas, Figure 5) the drug already presented in the previous paragraphs possesses a combined multichannel blocking effect. As mentioned above, vernakalant blocked in a similar range of concentration both I_Kur_ and I_Na_ channels [95,96], so the reported antiarrhythmic/antifibrillatory effect of the compound is also a combined atrial selective I_Kur_/I_Na_ blocking effect.

##### Atrial Acetylcholine-Sensitive Potassium Current (I_K,ACh_) Blockers

The acetylcholine activated inward rectifier K^+^ current, I_K,ACh_, is an another atrial-selective current. Its blockade is expected to exert a useful effect in vagally-induced atrial fibrillation. The I_K.ACh_ blockers may be real atrial selective therapeutic agents, since I_K,ACh_ is absent in the ventricles. There are many investigations demonstrating that I_K,ACh_ stimulation (via vagal stimulation) by reducing atrial APD/ERP increased sodium channel availability, and consequently creates re-entry substrate by increasing atrial repolarization dispersion. At faster frequencies, these re-entries may even form atrial rotors and may increase the time duration of the AF episodes [116]. As a consequence, we may state that vagus nerve activity plays an important role in initiation of paroxysmal AF [117,118], therefore, it was proposed that blockade of parasympathetic activity may have beneficial effects and could help to maintain SR. Consequently, selective I_K,ACh_ blockade may be a promising atrial selective therapeutic strategy (ARDA) [116,119].

**NIP-142** (Figure 5) and **NIP-151** (chemical structures not disclosed). The NIP-142 is a benzopyrane derivative compound, and was initially synthetized as highly selective I_K,A__C_h__ blocker. Experimental data demonstrated that accordingly with the hypothesis presented before, NIP-142 prevented the acetylcholine-induced AP shortening [120]. NIP-151 (a congener derivative) seems to be even more selective and more potent I_K,ACh_ blocker compound than NIP-142. NIP-151 significantly lengthened atrial ERP and prevented vagally- and aconitine-induced AF in an atrial selective manner in a dog model of AF [121].

##### Constitutively Active I_K,ACh_ Channels (I_K,Ach_Const_)

Recently, it has been published that in atrial myocardium originating from patients in permanent AF, acetylcholine-activated potassium channels are constitutively active (I_KA,Ch_Const_), namely they are opened without direct ligand stimulation [116,122]. These studies hypothesised that in long-term chronic AF, the constitutively active I_K,ACh_ current is one of main factors responsible for the triangularization and shortening of APD, thus making the atria susceptible for re-entry based tachyarrhythmias [116]. Conclusion of this observation is that selective blockade of I_KA,Ch_Const_ may have potent antiarrhythmic/antifibrillatory effects [116,120]. Because of the lack of selective I_KA,Ch_Const_ antagonists, this hypothesis could not be proved directly so far.

However, our recent study questioned this hypothesis. In an experimentally induced tachypaced dog model of permanent AF (ATR) we have revealed that the real amplitude of I_KA,Ch_Const_ does not seem to be large enough to contribute substantially to the shortening of APD and thus to the shortening of atrial ERP [13,123,124]. Therefore, we have proposed the following new concept [13]: under physiological condition, in vivo, “background” vagal stimulation is always present, thereby it is probable that the acetylcholine-activated potassium channels are active in atrial myocytes either in SR or in AF. In permanent AF, the I_KA,Ch_Const_ can also be activated, and its effect is added to the vagal nerve stimulated I_KA,Ch_, so these net outward currents can be large enough to cause atrial ERP/APD shortening. This hypothesis suggests that blockade of both basal I_K,ACh_ and I_KA,Ch_Const_ currents may prevent AF. Indeed, recently we have clearly demonstrated the presence of I_KA,Ch_Const_ in atrial cardiomyocytes isolated from ATR dogs, near of a clear presence of cholinergic activated I_K,ACh_ [13]. Selective blockade of these two combined currents with low concentration (18 and 56 µg/kg *iv.,* respectively) of **Tertiapin Q** (Figure 5) successfully prevented the experimentally induced AF in conscious ATR dogs [13,124]. Further experiments are performed recently to provide more evidence for our hypothesis. It remains questionable whether the results obtained in the dog heart can be directly translated to humans, since the newly developed compounds failed to prolong AERP and to reduce AF burden in humans [125,126].

#### 3.2.4. NCX Modulators

Under resting conditions, the Na^+^/Ca^2+^ exchanger (NCX) transports one intracellular Ca^2+^ ion for three extracellular sodium ions, resulting in net inward current. In depolarized cells, this bidirectional Na^+^/Ca^2+^ exchanger works in reverse mode; consequently it elevates the intracellular Ca^2+^ level and generates net outward current, which contributes to the shortening of the action potential. Rapid atrial rates caused by AF or rapid pacing favours the reverse mode of NCX (e.g., NCX1 activity). This may trigger AF by increasing the incidence of DAD arrhythmias. Based on this observation, it has been hypothesised that selective NCX blockade may prevent the formation of triggered arrhythmias, and consequently may prevent, reduce, or suppress AF, i.e., it was proposed that NCX blockers can be useful antiarrhythmic drugs [127,128,129]. However, lack of highly potent and selective NCX blockers made not possible direct verification of this plausible hypothesis. The first truly potent and selective blockers of the NCX were reported only after 2000.

**KB-R7943** (Kanebo, Tokyo, Japan, Figure 5) is a substance that inhibits preferably only the reverse mode of the NCX. Unfortunately, several studies revealed that the selectivity level of the compound is not high enough, i.e., at the same concentrations, the NCX blocking effect was also associated by a clear blocking potency on numerous other (I_to,_ I_K_, I_K1_, I_CaL_ and I_Na_) channels. Several studies reported that KB-R7943 prevented atrial ERP shortening caused by pacing-induced AF in anesthetized dogs, [130,131], but this effect of KB-R7943 cannot be attributed solely to its NCX blocking properties since as mentioned before, this molecule may also modulate the function of other ion channels.

**SEA0400** (Taisho Pharmaceutical, Tokyo, Japan, Figure 5) is a more selective NCX inhibitor than KB-R7943, and it was the most studied NCX blocker compound for several years. It was published that micromolar concentrations of SEA0400 effectively blocks NCX in both forward and reverse mode; however, it turned out that micromolar concentrations of SEA0400 effectively suppressed the L-type Ca current (I_CaL_) as well [131]. By applying SEA0400 as NCX blocker in human atrial tissue preparations, it was reported that the NCX current is significantly upregulated in AF as compared to SR [132]. Despite another study, which revealed that NCX blockade with SEA0400 may suppress ectopic automaticity in pulmonary veins [129], it is important to emphasize that these observations remain questionable because of the lack of trusted selective NCX blocking effect [128].

**ORM-10962** (Orion Pharmaceutical, Espoo, Finland), Figure 5) is a newly developed, and purportedly the most selective and highly potent NCX blocker. It has been published that a submicromolar concentration of ORM-10962 significantly reduced both the inward and outward NCX currents (reverse and forward mode of NCX). This molecule did not significantly change the main potassium currents involved in action potential repolarization (I_to_, I_Kr_, I_Ks_, I_K1_), the L-type Ca^2+^ current and the Na^+^/K^+^ pump. One µM ORM-10962 had no effect on the maximum rate of depolarization (dV/dt_max_) of action potential which indicates that fast inward Na^+^ current is unaffected. However, the amplitude of pharmacologically induced early and delayed afterdepolarizations was significantly decreased by ORM-10962 (3 and 10 μM) in a concentration-dependent manner [133]. These results suggest that ORM-10962 is the firstly reported highly selective NCX blocker [133].

In a recent study, the effect of combined ranolazine and SEA0400 and ORM-10103 compounds were investigated in an established experimental model of AF in rabbits. AF was induced by isoproterenol and acetylcholine. Combined ranolazine and NCX blocker experiments showed that the inducibility of AF was reduced in both groups by the additional perfusion with ranolazine and the selective NCX-inhibitors [134].

#### 3.2.5. Gap Junctions Modulators

Gap junctions are clusters of ion channels that provide connections and communication among cells, allowing the passage of ions and small molecules. They are composed of two hemichannels (connexons), while the connexon consists of six connexin proteins. Cardiac connexins have different isoforms (molecular weight of 30, 37, 40, 43 and 45 kDa). Human atria express particularly connexin 40, and connexin 43 [135,136]. Localization and function of connexins are governed by posttranslational modification and connexin phosphorylation [137]. Most of the gap junction channels are localized at the poles of cardiac cells [138] in close proximity to voltage gated sodium channels [139], while a smaller portion of gap junction channels can be found on the lateral side of cardiomyocytes. The consequence of this uneven distribution of gap junction channels is anisotropic conduction, namely the transversal conduction velocity is smaller than the longitudinal one [140]. Cell-to-cell coupling via gap junctions determines the conduction velocity and tend to smooth out the beat-to beat variations in action potential duration, which can affect refractoriness.

Under pathological conditions such as ischaemia, gap junction channels close, which slows conduction and reduces action potential duration resulting in increased beat-to-beat variability of action potential duration and dispersion of refractory period. The dispersion of action potential duration is pronounced in the ischaemic area especially in the border zone [141,142]. These may result in a meandering activation pathway, conduction block, and re-entry arrhythmias [143,144,145]. Conduction disturbances can be caused by fibrosis as well, since it deteriorates side-to-side electrical coupling of atrial cells [146], therefore fibrotic border zone may become a substrate for re-entrant arrhythmia [147].

Electrical and structural remodelling caused by permanent AF accompanies changes in the atrial intercalated discs. Alteration of junctional complexes of intercalated discs like fascia adherens junctions (*N*-cadherin), desmosomes (desmoplakin), and the gap junction proteins (connexins) was described [147]. Remodelling associated with AF causes heterogeneous spatial distribution of gap junctions. Although the published results are inconsistent concerning CX40 and CX43 expression in the atria of patients with AF, however, most of the results demonstrate the redistribution of gap junctions from the poles of cardiomyocytes to the lateral sides of these cells. This heterogeneous gap junction distribution seems independent of AF aetiology [148,149,150]. The heterogeneous distribution of gap junctions may lead to increased dispersion in refractoriness, to altered conduction and non-uniform anisotropic characteristics that can facilitate re-entrant circuits [151].

In some models of AF (like ischemia and mitral valve disease-related AF), the administration of specific gap junction modulators that prevent closure of cardiac gap junctions may possess antiarrhythmic effect against AF [152,153,154,155]. In the porcine model of burst pacing-induced AF, the atrial overexpression of connexin 40 and connexin 43 preserved conduction velocity and prevented sustained AF [156]. However, these specific gap junction modulators may have minor effects when the altered conduction and non-uniform anisotropic characteristics is caused by the heterogeneous gap junction distribution [157,158,159]. In bovine atrium an endogenous antiarrhythmic peptide (AAP) was identified, which acts on the gap junction [160]. Several derivatives of AAP were synthetized that enhance gap junction conduction like AAP10, ZP123 and GAP-134 [161,162,163].

**AAP10** (Figure 5) was one of the first studied antiarrhythmic coupling peptides [161]. AAP10 improved both metabolic and electrical coupling on guinea pig and rat ventricular cells and in atrial cells [164,165,166]. In HeLa cells expressing Cx43, Cx40, AAP10 remodelled coupling via Cx43 and to a lesser extent Cx40 gap junction channels, which was followed by enhanced Cx43 or Cx40 protein expression [166,167].

**Rotigaptide** (**GAP-486**, **ZP123**, Figure 5) is a synthetic AAP analogue. The difference between AAP10 and GAP-486 is that in rotigaptide, the d-isomers have been substituted for l-isomers. Rotigaptide was the first molecule developed to protect against gap junction closure. It selectively enhances gap junction conductance and cell-to-cell coupling without affecting the function of other ion channels. Using the acute atrial stretch model of atrial fibrillation it was reported that the rotigaptide treatment effectively reduced inducibility of the sustained atrial fibrillation [168]. It has been reported that rotigaptide effectively attenuates ventricular arrhythmogenesis and atrial conduction velocity slowing while it has no effect on control [157,158,169]. In rats and dogs, rotigaptide reduced infarct size following myocardial infarction [170], and this peptide prevented Cx43 dephosphorylation during ischemia [171]. Subchronic exposure (12–24 h) to rotigaptide enhanced Cx43 expression [172], however, Clarke et al. did not observe increase in Cx43 expression but increased gap junction communication was reported by ZP123 [173]. ZP123 was administered intravenously during Phase II clinical trials, which were conducted on myocardial ischaemic patients with non-lethal ventricular arrhythmias [174]. In spite of promising results, the development of ZP123 was terminated [174].

**Danegaptide** (GAP 134, ZP1609, Figure 5) is a small dipeptide analogue of rotigaptide with oral bioavailability [163]. Using rat atrial muscle strips, it was demonstrated that administration of GAP 134 prevented conduction slowing elicited by metabolic stress. This emphasizes the GAP 134 ability to maintain cell-to-cell coupling [175]. In a whole-animal model, in the canine model of postoperative AF GAP 134 increased conduction velocity and significantly reduced AF duration without affecting the refractory period [175]. Based on these results, it was suggested to use GAP 134 as a preventive treatment for post-operative AF [175]. In a dog model of pacing-induced atrial myopathy 14 days GAP 134 treatment did not change Cx43 and Cx40 mRNA levels and the spatial distribution of Cx43. However, GAP 134 attenuated AF vulnerability in paced dogs with minimal left atrial dilation, although it had no effect on AF inducibility in animals with marked left atrial dilation. This suggests that in case of marked structural remodelling (profound fibrosis), gap junction enhancers cannot affect AF inducibility [159].

In a recent clinical study, the effect of danegaptide treatment was evaluated in patients with ST-segment elevation myocardial infarction. However, we must note that unfortunately, danegaptide treatment did not improve myocardial salvage of these patients [176].

**PQ1** (6-Methoxy-8-[(3-amionpropyl) amino]-4-methyl-5-(3-trifluoromethyl-phen loxy) quinolone, Figure 5) is the most effective antiproliferative agent among synthetic quinoline analogues [177]. PQ1-induced cytotoxicity can be related to its enhancing effect on gap junction communication, namely PQ1 increases activity of existing Cx43, shifts the expression and enabling of Cx43 to be localized to the plasma membrane [178]. Indeed, in T47D breast cancer cells. PQ1 caused at least an eight-fold increase in gap junction activity and decreased growth [177,179]. However, it has been also demonstrated that PQ1 induces apoptosis via caspase 8 and 9, as well [179]. Oral bioavailability studies prove that PQ1 has a low toxicity to normal healthy tissue [177]. Chang et al. studied the effects of PQ1 on sinoatrial node and pulmonary veins [180]. As it was detailed above, the conduction block between sinoatrial node and pulmonary veins is proarrhythmic and increases the risk of AF occurrence [181]. Chang et al. also reported that in pulmonary vein-sinoatrial node preparation, the application of the gap junction inhibitor heptanol reduced spontaneous activity of sinoatrial node while pulmonary vein activity was unsuppressed and burst firings were observed. The administration of gap junction enhancer PQ1 eliminated pulmonary vein burst firings and prevented pulmonary vein arrhythmogenesis [180].

In summary, it is generally accepted that altered gap junction channel function contributes to the development of AF; therefore, molecules acting on gap junction channels can be effective pharmacological targets against AF. However, further detailed investigations are required to explore their potential in AF treatment.

### 3.3. Other Possible Ion Channel Targets for Novel Antiarrhythmic Drugs

Beside the well-known ion channels the two pore-domain potassium channels (K2P [182]), small-conductance calcium-activated potassium (SK) channels [183], calcium activated K^+^-channels [184], transient receptor channels (TRP [185]), mechanosensitive and stretch activated channels [186] contribute to the development of cardiac action potential; therefore, these ion channels can be useful target for antiarrhythmic exploitation as well. However, based on the results of the relatively small number of investigations there are no or only a very few promising findings suggesting that molecules acting on these ion channels can be useful in preventing AF.

### 3.4. Non Ion-Channel Blockers—Upstream Therapy of AF

The common feature of the above detailed therapeutic possibilities is that they are related to the alteration of ion channel or gap junctional channel functions. However, there is a rapid development of the so-called non-ion channel approaches. The goal of these approaches is to reverse, or to reduce structural remodelling caused by oxidative stress and inflammation. These approaches are called as “upstream therapies” [187,188].

As previously detailed, structural remodelling is promoted by pathophysiological conditions like inflammation and oxidative injury, fibroblast proliferation, accumulation and/or redistribution of collagen fibres, cardiac chamber dilation, and hypertrophy. Proarrhythmic effects of atrial remodelling are associated with AF because they are generally related to conduction disturbances, which promote re-entrant type arrhythmias. Both experimental and clinical studies have demonstrated that these so-called “upstream therapy” drugs may affect structural remodelling, inflammation, and/or oxidative stress and this way they may decrease the occurrence of AF. The occurrence of AF can be reduced by inhibition of angiotensin-converting enzyme, administration of angiotensin II receptor blockers, and statins as well [189,190]. In contrast, other studies questioned the efficacy of these therapies in AF [191]. However, it is necessary to emphasize that the use of statins is significantly associated with a reduced risk of AF in patients with sinus rhythm [192]. The highest benefit was seen for the prevention of postoperative atrial fibrillation and in secondary prevention of atrial fibrillation [193,194]. Unfortunately, the precise role and contribution of oxidative stress and inflammation to the development of AF is still not fully understood [41,195,196].

## 4. Conclusions

During the past ten years, a large number of papers were published concerning the pathophysiology of AF, which help to understand the mechanisms and consequences of the development of AF and the underlying atrial remodelling. Both ion channel and non-ion-channel-related therapeutic approaches are in use to prevent, suppress or protect against AF. However, the currently available pharmacological treatments are far from being ideal. Based on the recent knowledge of the pathophysiology of AF new antiarrhythmic drugs were developed which have high affinity to the atrial myocardium and may reduce the risk of AF. Nevertheless, it is also important to produce new molecules capable of slowing down or preventing the recurrence of AF. Numerous publications have demonstrated the efficacy of the recently developed molecules, which have been extensively studied. However, only larger-scale prospective trials will prove if the use of these antiarrhythmic compounds will result in improved prognosis of patients with AF.

## Figures and Tables

**Table 1:** Summary of the new drugs and investigational compounds developed for treating AF presented in this review.

	Name of Compounds or Drugs	Major Effects	Clinical Studies
Further development and improvement of existing antiarrhythmic drugs or compounds	Azimilide (FDA approval)	primarily I_Kr_ and I_Ks_ blocker but additionally blocks I_CaL_ and I_Na_ (multi-channel blocker)	ALIVE, A-STAR, A-COMET I and II Studies
	HMR-1556	highly selective I_Ks_ blocker	not performed
	AZD7009	primarily I_Kr_ and I_Na_ blocker, but additionally blocks I_to_, I_Kur_ and I_Ks_ (multi-channel blocker)	small single centre clinical trial
	Dronedarone (FDA approval)	Amiodarone-like multichannel blocker (I_Na_, I_Ca_, I_Kr_ blocker)	ADONIS, ATHENA, EURIDIS etc.
	Tedisamil	multichannel blocker (I_Na_, I_to_, I_Kr_, I_Ks_, I_KATP_, blocker)	small single centre clinical trial
Atrial selective therapeutic agents (existing drugs or investigational compounds)	AVE0118	primarily I_Kur_, I_to_ and I_K,ACh_ blocker	not carried out
	XEN-D0101	highly selective I_Kur_ blocker	small single centre clinical trial
	DP01	highly selective I_Kur_ blocker	not carried out
	Vernakalant	primarily I_Kr_ and I_Na_ blocker, but additionally blocks I_to_, I_Na_, I_Kr_ and I_Ks_ (multichannel blocker)	AVRO
	Ranolazine (FDA approval)	primarily I_Naf_ and I_NaL_, and I_Kr_ blocker, but additionally blocks I_CaL_ and I_Ks_ (multichannel blocker)	MERLIN-TIMI
	NIP-142, NIP-152	highly selective I_K,ACh_ blockers	not carried out
	Tertiapin Q	highly selective I_K,ACh_ blocker	not carried out
NCX modulators	KB-R7943	initially developed as selective NCX blocker, but additionally blocks I_to,_ I_K_, I_K1_, I_Na_, and I_CaL_	not carried out
	SEA-0400	selective NCX blocker, but additionally blocks I_CaL_	not carried out
	ORM-10962	highly selective and potent NCX blocker	not carried out
Gap-junction modulators	AAP10	selective gap junction enhancer peptide	not carried out
	Rotigaptide	selective gap junction enhancer peptide	ClinicalTrials.gov Identifier NCT00137332
	Danegaptide (GAP-134)	selective gap junction enhancer peptide	ClinicalTrials.gov Identifier NCT00510029, NCT00543946, and NCT00783341
	PQ1	selective gap junction enhancer	not carried out

## Data Availability

Data sharing not applicable.

## Abbreviations

AF	atrial fibrillation
AFlu	atrial flutter
AERP	atrial effective refractory period
APD	action potential duration
ARDA	atrial repolarizing delayed agents
ATR	experimentally induced tachypaced dog model of permanent AF
CICR	Ca^2+^-induced Ca^2+^-release
CVPT	catecholaminergic polymorphic ventricular tachycardia
DAD	delayed afterdepolarization
EAD	early afterdepolarization
FDA	Food and Drug Administration
I_K_,_Ach_	acetylcholine sensitive potassium current
I_K,Ach_Const_	constitutively active acetylcholine sensitive potassium current (I_K,Ach_)
I_Ca,L_	L-type calcium current
I_K1_	inward rectifier potassium current
I_KATP_	ATP-sensitive potassium current
I_Kr_	rapid component of the delayed rectifier potassium current
I_Ks_	slow component of the delayed rectifier potassium current
I_Kur_	ultra-rapid component of the delayed rectifier potassium current
I_to_	transient outward potassium current
I_Na_	fast inward sodium current
I_NaL_	late sodium current
I_NCX_	sodium-calcium (Na^+^/Ca^2+^) exchanger current (NCX)
miRNA	small single-stranded non-coding RNA)
NSAID	non-steroidal anti-inflammatory drugs
PVs	left upper pulmonary vein
RAAS	renin-angiotensin-aldosterone system
SMAD	Mothers Against Decapentaplegic is a protein from the SMAD family
SR	sinus rhythm
TGF	transforming growth factor

## References

[1] Beyerbach D.M., Zipes D.P. (2004). Mortality as an endpoint in atrial fibrillation. Heart Rhythm..

[2] Nattel S., Opie L.H. (2006). Controversies in atrial fibrillation. Lancet.

[3] Go A.S., Hylek E.M., Phillips K.A., Chang Y., Henault L.E., Selby J.V., Singer D.E. (2001). Prevalence of diagnosed AF in adults: National implications for rhythm management and stroke prevention: The anTicoagulation and Risk Factors in AF (ATRIA) Study. JAMA.

[4] A Wolf P., Abbott R.D., Kannel W.B. (1987). Atrial fibrillation: A major contributor to stroke in the elderly. The Framingham Study. Arch. Intern. Med..

[5] Nattel S., Maguy A., Le Bouter S., Yeh Y.-H. (2007). Arrhythmogenic Ion-Channel Remodeling in the Heart: Heart Failure, Myocardial Infarction, and Atrial Fibrillation. Physiol. Rev..

[6] Camm A.J., Lip G.Y., De Caterina R., Savelieva I., Atar D. (2012). Focused update of ESC Guidelines for the management of atrial fibrillation: An update of the 2010 guidelines for the management of atrial fibrillation—Developed with the special contribution of the European Heart Rhythm Association. Europace.

[7] Allessie M.A., I Bonke F., Schopman F.J. (1977). Circus movement in rabbit atrial muscle as a mechanism of tachycardia. III. The “leading circle” concept: A new model of circus movement in cardiac tissue without the involvement of an anatomical obstacle. Circ. Res..

[8] Ravens U., Poulet C., Wettwer E., Knaut M. (2013). Atrial selectivity of antiarrhythmic drugs. J. Physiol..

[9] Pandit S.V., Jalife J. (2013). Rotors and the Dynamics of Cardiac Fibrillation. Circ. Res..

[10] Allessie M., Ausma J., Schotten U. (2002). Electrical, contractile and structural remodeling during atrial fibrillation. Cardiovasc. Res..

[11] Dobrev D., Ravens U. (2003). Remodeling of cardiomyocyte ion channels in human atrial fibrillation. Basic Res. Cardiol..

[12] Nattel S., Burstein B., Dobrev D. (2008). Atrial remodeling and atrial fibrillation: Mechanisms and implications. Circ. Arrhythmia Electrophysiol..

[13] Jost N., Kohajda Z., Kristof A., Kovacs P., Juhasz V., Kiss L., Varro A., Virag L., Baczko I. (2011). Atrial remodeling and novel pharmacological strategies for an-tiarrhythmic therapy in atrial fibrillation. Curr. Med. Chem..

[14] Nattel S., Dobrev D. (2012). The multidimensional role of calcium in atrial fibrillation pathophysiology: Mechanistic insights and therapeutic opportunities. Eur. Heart J..

[15] Healey J.S., Israel C.W., Connolly S.J., Hohnloser S.H., Nair G.M., Divakaramenon S., Capucci A., Van Gelder I.C., Lau C.P., Gold M.R. (2012). Relevance of electrical remodeling in human atrial fibrillation: Results of the asymptomatic atrial fibrillation and stroke evaluation in pacemaker patients and the atrial fibrillation reduction atrial pacing trial mechanisms of atrial fibrillation study. Circ. Arrhythm Electrophysiol..

[16] Darbar D. (2008). Genetics of atrial fibrillation: Rare mutations, common polymorphisms, and clinical relevance. Heart Rhythm.

[17] Ellinor P.T., Yi B.A., MacRae C.A. (2008). Genetics of atrial fibrillation. Med. Clin. N. Am..

[18] Wolff L. (1943). Familial Auricular Fibrillation. N. Engl. J. Med..

[19] Fox C.S., Parise H., D’Agostino R.B.S. (2004). Parental AF as a risk factor for AF in offspring. JAMA.

[20] Pastori D., Menichelli D., Lip G.Y.H., Sciacqua A., Violi F., Pignatelli P., ATHERO-AF Study Group (2020) (2004). Family history of atrial fibrillation and risk of cardiovascular events: A multicenter prospective cohort study. Circ. Arrhythm Electrophysiol..

[21] Hucker W.J., Hanley A., Ellinor P.T. (2017). Improving atrial fibrillation therapy: Is there a gene for that?. J. Am. Coll. Cardiol..

[22] Cheng H., Lederer W.J. (2008). Calcium sparks. Physiol. Rev..

[23] Greiser M., Lederer W.J., Schotten U. (2010). Alterations of atrial Ca2+ handling as cause and consequence of atrial fibrillation. Cardiovasc. Res..

[24] Greiser M., Schotten U. (2013). Dynamic remodeling of intracellular Ca2+ signaling during atrial fibrillation. J. Mol. Cell. Cardiol..

[25] Voigt N., Dobrev D. (2012). Cellular and molecular correlates of ectopic activity in patients with atrial fibrillation. Europace.

[26] Christ T. (2014). Atrial-selective Antiarrhythmic Activity by Vernakalant Fact or Fiction?. J. Cardiovasc. Pharmacol..

[27] Berk E., Christ T., Schwarz S., Ravens U., Knaut M., Kaumann A.J. (2016). In permanent atrial fibrillation, PDE3 reduces force responses to 5-HT, but PDE3 and PDE4 do not cause the blunting of atrial arrhythmias. Br. J. Pharmacol..

[28] Yue L., Feng J., Gaspo R., Li G.-R., Wang Z., Nattel S. (1997). Ionic Remodeling Underlying Action Potential Changes in a Canine Model of Atrial Fibrillation. Circ. Res..

[29] Sun H., Gaspo R., Leblanc N., Nattel S. (1998). Cellular mechanisms of atrial contractile dysfunction caused by sustained atrial tachycardia. Circulation.

[30] Schotten U., Duytschaever M., Ausma J., Eijsbouts S., Neuberger H.-R., Allessie M. (2003). Electrical and Contractile Remodeling During the First Days of Atrial Fibrillation Go Hand in Hand. Circulation.

[31] Schotten U., Neuberger H.-R., Allessie M.A. (2003). The role of atrial dilatation in the domestication of atrial fibrillation. Prog. Biophys. Mol. Biol..

[32] Kostin S., Klein G., Szalay Z., Hein S., Bauer E.P., Schaper J. (2002). Structural correlate of atrial fibrillation in human patients. Cardiovasc. Res..

[33] Burstein B., Nattel S. (2008). Atrial Fibrosis: Mechanisms and Clinical Relevance in Atrial Fibrillation. J. Am. Coll. Cardiol..

[34] Smit M.D., Moes M.L., Maass A.H., Achekar I.D., Van Geel P.P., Hillege H.L., Van Veldhuisen D.J., Van Gelder I.C. (2012). The importance of whether atrial fibrillation or heart failure develops first. Eur. J. Heart Fail..

[35] Pellman J., Lyon R.C., Sheikh F. (2010). Extracellular matrix remodeling in atrial fibrosis: Mechanisms and implications in atrial fibrillation. J. Mol. Cell. Cardiol..

[36] Schmidt M., Christiansen C., Mehnert F., Rothman K., Sørensen H.T. (2011). Non-steroidal anti-inflammatory drug use and risk of atrial fibrillation or flutter: Population based case-control study. BMJ.

[37] Cardin S., Guasch E., Luo X., Naud P., Le Quang K., Shi Y., Tardif J.-C., Comtois P., Nattel S. (2012). Role for MicroRNA-21 in Atrial Profibrillatory Fibrotic Remodeling Associated with Experimental Postinfarction Heart Failure. Circ. Arrhythmia Electrophysiol..

[38] Wang Z., Lu Y., Yang B. (2010). MicroRNAs and atrial fibrillation: New fundamentals. Cardiovasc. Res..

[39] Shan H., Zhang Y., Lu Y., Pan Z., Cai B., Wang N., Xuelian L., Feng T., Hong Y., Yang B. (2009). Downregulation of miR-133 and miR-590 contributes to nicotine-induced atrial remodelling in canines. Cardiovasc. Res..

[40] Ling T.-Y., Wang X.-L., Chai Q., Lau T.-W., Koestler C.M., Park S.J., Daly R.C., Greason K.L., Jen J., Wu L.-Q. (2013). Regulation of the SK3 channel by microRNA-499—Potential role in atrial fibrillation. Heart Rhythm.

[41] Lip G.Y., Fauchier L., Freedman S.B., Van Gelder I., Natale A., Gianni C., Nattel S., Potpara T., Rienstra M., Tse H.F. (2016). Atrial fibrillation. Nat Rev Dis Primers.

[42] Jurkiewicz N.K., Sanguinetti M.C. (1993). Rate-dependent prolongation of cardiac action potentials by a methanesulfonanilide class III antiarrhythmic agent. Specific block of rapidly activating delayed rectifier K+ current by dofetilide. Circ. Res..

[43] Salata J.J., Brooks R.R. (1997). Pharmacology of Azimilide Dihydrochloride (NE-10064), A Class III Antiarrhythmic Agent. Cardiovasc. Drug Rev..

[44] Light P. (2000). Azimilide (Procter & Gamble). Drugs Investig. Drugs J..

[45] Karam R., Marcello S., Brooks R.R., E Corey A., Moore A. (1998). Azimilide Dihydrochloride, a Novel Antiarrhythmic Agent. Am. J. Cardiol..

[46] Connolly S.J., Schnell D.J., Page R.L., E Wilkinson W., Marcello S.R., Pritchett E.L. (2001). Dose-response relations of azimilide in the management of symptomatic, recurrent, atrial fibrillation. Am. J. Cardiol..

[47] Camm A.J., Pratt C.M., Schwartz P.J., Al-Khalidi H.R., Spyt M., Holroyde M.J., Karam R., Sonnenblick E.H., Brum J.M. (2001). Azimilide Post Infarct Survival Evaluation (ALIVE): Azimilide does not affect mortality in post-myocardial infarction patients. Circulation.

[48] Camm A.J., Pratt C.M., Schwartz P.J., Al-Khalidi H.R., Spyt M.J., Holroyde M.J., Karam R., Sonnenblick E.H., Brum J.M. (2004). Azimilide post Infarct surVival Evaluation (ALIVE) Investigators. Mortality in patients after a recent myocardial infarction: A random-ized, placebo-controlled trial of azimilide using heart rate variability for risk stratification. Circulation.

[49] Pratt C.M., Singh S.N., Al-Khalidi H.R., Brum J.M., Holroyde M.J., Marcello S.R., Schwartz P.J., Camm A. (2004). The efficacy of azimilide in the treatment of atrial fibrillation in the presence of left ventricular systolic dysfunction: Results from the Azimilide Postinfarct Survival Evaluation (ALIVE) trial. J. Am. Coll. Cardiol..

[50] So P.P.-S., Backx P.H., Dorian P. (2008). Slow delayed rectifier K+ current block by HMR 1556 increases dispersion of repolarization and promotes Torsades de Pointes in rabbit ventricles. Br. J. Pharmacol..

[51] Nakashima H., Gerlach U., Schmidt D., Nattel S. (2004). In vivo electrophysiological effects of a selective slow delayed-rectifier potassium channel blocker in anesthetized dogs: Potential insights into class III actions. Cardiovasc. Res..

[52] Goldstein R.N., Khrestian C., Carlsson L., Waldo A.L. (2004). AZD7009: A new antiarrhythmic drug with predominant effects on the atria effectively terminates and prevents reinduction of atrial fibrillation and flutter in the sterile pericarditis model. J. Cardiovasc. Electrophysiol..

[53] Persson F., Carlsson L., Duker G., Jacobson I. (2005). Blocking Characteristics of hKv1.5 and hKv4.3/hKChIP2.2 After Administration of the Novel Antiarrhythmic Compound AZD7009. J. Cardiovasc. Pharmacol..

[54] Wu Y., Carlsson L., Liu T., Kowey P.R., Yan G.-X. (2005). Assessment of the Proarrhythmic Potential of the Novel Antiarrhythmic Agent AZD7009 and Dofetilide in Experimental Models of Torsades De Pointes. J. Cardiovasc. Electrophysiol..

[55] Crijns H.J., Van Gelder I.C., Walfridsson H., Kulakowski P., Rónaszéki A., Dedek V., Malm A., Almgren O. (2006). Safe and effective conversion of persistent atrial fibrillation to sinus rhythm by intravenous AZD7009. Heart Rhythm.

[56] Aunes-Jansson M., Edvardsson N., Stridh M., Sörnmo L., Frison L., Berggren A. (2013). Decrease of the atrial fibrillatory rate, in-creased organization of the atrial rhythm and termination of atrial fibrillation by AZD7009. J. Electrocardiol..

[57] Aunes M., Egstrup K., Frison L., Berggren A., Stridh M., Sörnmo L., Edvardsson N. (2014). Rapid slowing of the atrial fibrillatory rate after administration of AZD7009 predicts conversion of atrial fibrillation. J. Electrocardiol..

[58] Hondeghem L.M., Snyders D. (1990). Class III antiarrhythmic agents have a lot of potential but a long way to go. Reduced effectiveness and dangers of reverse use dependence. Circulation.

[59] Hondeghem L.M., Katzung B.G. (1984). Antiarrhythmic agents: The modulated receptor mechanism of action of sodium and calcium channel-blocking drugs. Annu. Rev. Pharmacol. Toxicol..

[60] Aimond F., Beck L., Gautier P., Chérif O.K., Davy J.M., Lorente P., Nisato D., Vassort G. (2000). Cellular and in vivo electrophysio-logical effects of dronedarone in normal and postmyocardial infarcted rats. J. Pharmacol. Exp. Ther..

[61] Varró A., Takács J., Németh M., Hála O., Virág L., Iost N., Baláti B., Ágoston M., Vereckei A., Pastor G. (2001). Electrophysiological effects of dronedarone (SR 33589), a noniodinated amiodarone derivative in the canine heart: Comparison with amiodarone. Br. J. Pharmacol..

[62] Singh B.N., Connolly S.J., Crijns H.J., Roy D., Kowey P.R., Capucci A., Radzik D., Aliot E.M., Hohnloser S.H. (2007). Dronedarone for Maintenance of Sinus Rhythm in Atrial Fibrillation or Flutter. N. Engl. J. Med..

[63] Hoy S.M., Kean S.J. (2009). Dronedarone. Drugs.

[64] Torp-Pedersen C., Crijns H.J., Gaudin C., Page R.L., Connolly S.J., Hohnloser S.H., ATHENA Investigators (2011). Impact of dronedarone on hospitalization burden in patients with atrial fibrillation: Results from the ATHENA study. Europace.

[65] Burashnikov A., Belardinelli L., Antzelevitch C. (2010). Acute dronedarone is inferior to amiodarone in terminating and preventing atrial fibrillation in canine atria. Heart Rhythm.

[66] Franz M.R., Singh S.N. (2010). Amiodarone and dronedarone: The worker bee and the drone?. Heart Rhythm.

[67] Joghetaei N., Weirich G., Huber W., Büchler P., Estner H. (2011). Acute Liver Failure Associated with Dronedarone. Circ. Arrhythmia Electrophysiol..

[68] Boriani G., Blomström-Lundqvist C., Hohnloser S.H., Bergfeldt L., Botto G.L., Capucci A., Lozano I.F., Goette A., Israel C.W., Merino J.L. (2019). Safety and efficacy of dronedarone from clinical trials to real-world evidence: Implications for its use in atrial fibrillation. Europace.

[69] Jost N., Virag L., Hala O., Varro A., Thormahlen D., Papp J.G. (2004). Effect of the Antifibrillatory Compound Tedisamil (KC-8857) on Transmembrane Currents in Mammalian Ventricular Myocytes. Curr. Med. Chem..

[70] Beatch G.N., Abraham S., MacLeod B.A., Yoshida N.R., Walker M. (1991). Antiarrhythmic properties of tedisamil (KC8857), a putative transient outward K+ current blocker. Br. J. Pharmacol..

[71] Flores N.A. (2001). Tedisamil (Solvay). Curr. Opin. Investig. Drugs.

[72] Barrett T.D., Hennan J.K., Fischbach P.S., O’Neill B.P., Driscoll E.M., Lucchesi B.R. (2001). Tedisamil and dofetilide-induced torsades de pointes, rate and potassium dependence. Br. J. Pharmacol..

[73] Fischbach P.S., Johnston P.V., Friedrichs G.S., Lucchesi B.R. (1999). Tedisamil in a Chronic Canine Model of Atrial Flutter. J. Cardiovasc. Pharmacol..

[74] Fischbach P.S., Barrett T.D., Goyal R., Tran B.C., Syed Z.A., Hennan J.K., Lucchesi B.R. (2001). Conversion of atrial fibrillation by the experi-mental antiarrhythmic drug tedisamil in two canine models. J. Cardiovasc. Electrophysiol..

[75] Hohnloser S.H., Dorian P., Straub M., Beckmann K., Kowey P. (2004). Safety and efficacy of intravenously administered tedisamil for rapid conversion of recent-onset atrial fibrillation or atrial flutter. J. Am. Coll. Cardiol..

[76] (2007). Drugs.com. https://www.drugs.com/history/pulzium.html.

[77] Roden D. (2008). Cardiology Today. FDA Panel Votes against Recommendation for Tedisamil. https://www.healio.com/news/cardiology/20120225/fda-panel-votes-against-recommendation-for-tedisamil.

[78] Wang Z., Fermini B., Nattel S. (1993). Sustained depolarization-induced outward current in human atrial myocytes. Evidence for a novel delayed rectifier K+ current similar to Kv1.5 cloned channel currents. Circ. Res..

[79] Amos G.J., Wettwer E., Metzger F., Li Q., Himmel H.M., Ravens U. (1996). Differences between outward currents of human atrial and subepicardial ventricular myocytes. J. Physiol..

[80] Wettwer E., Hála O., Christ T., Heubach J.F., Dobrev D., Knaut M., Varró A., Ravens U. (2004). Role of IKur in in controlling action potential shape and contractility in the human atrium: Influence of chronic atrial fibrillation. Circulation.

[81] Wirth K.J., Paehler T., Rosenstein B., Knobloch K., Maier T., Frenzel J., Brendel J., Busch A.E., Bleich M. (2003). Atrial effects of the novel K+-channel-blocker AVE0118 in anesthetized pigs. Cardiovasc. Res..

[82] Christ T., Wettwer E., Voigt N., Hála O., Radicke S., Matschke K., Varró A., Dobrev D., Ravens U. (2008). Pathology-specific effects of the IKur/Ito/IK,ACh blocker AVE0118 on ion channels in human chronic atrial fibrillation. Br. J. Pharmacol..

[83] Oros A., Volders P.G., Beekman J.D., van der Nagel T., Vos M.A. (2006). Atrial-specific drug AVE0118 is free of torsades de pointes in anesthetized dogs with chronic complete atrioventricular block. Heart Rhythm.

[84] De Haan S., Greiser M., Harks E., Blaauw Y., van Hunnik A., Verheule S., Allessie M., Schotten U. (2006). AVE0118, blocker of the transient outward current (I(to)) and ultrarapid delayed rectifier current (I(Kur)), fully restores atrial contractility after cardio-version of atrial fibrillation in the goat. Circulation.

[85] Blaauw Y., Schotten U., van Hunnik A., Neuberger H.R., Allessie M.A. (2007). Cardioversion of persistent atrial fibrillation by a combination of atrial specific and non-specific class III drugs in the goat. Cardiovasc. Res..

[86] Wirth K.J., Steinmeyer K., Ruetten H. (2013). Sensitization of upper airway mechanoreceptors as a new pharmacologic principle to treat obstructive sleep apnea: Investigations with AVE0118 in anesthetized pigs. Sleep.

[87] Rivard L., Shiroshita-Takeshita A., Maltais C., Ford J., Pinnock R., Madge D., Nattel S. (2005). Electrophysiological and atrial anti-arrhythmic effects of a novel IKur/Kv1.5 blocker in dogs. Heart Rhythm.

[88] Shiroshita-Takeshita A., Ford J., Madge D., Pinnock R., Nattel S. (2006). Electrophysiological and atrial antiarrhythmic effects of a novel IKur/Kv1.5 blocker in dogs with atrial tachycardia remodeling. Heart Rhythm.

[89] Ford J., Milnes J., Wettwer E., Christ T., Rogers M., Sutton K., Madge D., Virág L., Jost N., Horváth Z. (2013). Human electrophysiological and pharmacological properties of XEN-D0101: A novel atrial selective Kv1.5/IKur inhibitor. J. Cardiovasc. Pharmacol..

[90] Ford J., Milnes J., El Haou S., Wettwer E., Loose S., Matschke K., Tyl B., Round P., Ravens U. (2016). The positive frequency-dependent electrophysiological effects of the IKur inhibitor XEN-D0103 are desirable for the treatment of atrial fibrillation. Heart Rhythm.

[91] Shunmugam S.R., Sugihara C., Freemantle N., Round P., Furniss S., Sulke N. (2018). A double-blind, randomised, placebo-controlled, cross-over study assessing the use of XEN-D0103 in patients with paroxysmal atrial fibrillation and implanted pacemakers al-lowing continuous beat-to-beat monitoring of drug efficacy. J. Interv. Card. Electrophysiol..

[92] Stump G.L., Wallace A.A., Regan C.P., Lynch J.J. (2005). In vivo antiarrhythmic and cardiac electrophysiologic effects of a novel diphenylphosphine oxide IKur blocker (2-isopropyl-5-methylcyclohexyl) diphenylphosphine oxide. J. Pharmacol. Exp. Ther..

[93] Lagrutta A., Wang J., Fermini B., Salata J.J. (2006). Novel, potent inhibitors of human Kv1.5 K+ channels and ultrarapidly activating delayed rectifier potassium current. J. Pharmacol. Exp. Ther..

[94] McIntyre W.F., Healey J.S., Bhatnagar A.K., Wang P., Gordon J.A., Baranchuk A., Deif B., Whitlock R.P., Belley-Côté É.P. (2019). Vernakalant for cardioversion of recent-onset atrial fibrillation: A systematic review and meta-analysis. Europace.

[95] Fedida D., Orth P.M., Chen J.Y., Lin S., Plouvier B., Jung G., Ezrin A.M., Beatch G.N. (2005). The mechanism of atrial antiarrhythmic action of RSD1235. J. Cardiovasc. Electrophysiol..

[96] Wettwer E., Christ T., Endig S., Rozmaritsa N., Matschke K., Lynch J.J., Pourrier M., Gibson J.K., Fedida D., Knaut M. (2013). The new antiarrhythmic drug vernakalant: Ex vivo study of human atrial tissue from sinus rhythm and chronic atrial fibrillation. Cardiovasc. Res..

[97] Burashnikov A., Pourrier M., Gibson J.K., Lynch J.J., Antzelevitch C. (2012). Rate-dependent effects of vernakalant in the isolated non-remodeled canine left atria are primarily due to block of the sodium channel: Comparison with ranolazine and dl-sotalol. Circ. Arrhythm Electrophysiol..

[98] Dobrev D., Hamad B., Kirkpatrick P. (2010). Vernakalant. Nat. Rev. Drug Discov..

[99] Van Middendorp L.B., Strik M., Houthuizen P., Kuiper M., Maessen J.G., Auricchio A., Prinzen F.W. (2014). Electrophysiological and haemodynamic effects of vernakalant and flecainide in dyssynchronous canine hearts. Europace.

[100] Camm A.J., Capucci A., Hohnloser S.H., Torp-Pedersen C., Van Gelder I.C., Mangal B., Beatch G., AVRO Investigators (2011). A randomized active-controlled study comparing the efficacy and safety of vernakalant to amiodarone in recent-onset atrial fi-brillation. J. Am. Coll. Cardiol..

[101] Cialdella P., Pedicino D., Santangeli P. (2011). Novel Agents for the Acute Conversion of Atrial Fibrillation: Focus on Vernakalant. Recent Pat. Cardiovasc. Drug Discov..

[102] Simon A., Niederdoeckl J., Janata K., Spiel A.O., Schuetz N., Schnaubelt S., Herkner H., Cacioppo F., Laggner A.N., Domanovits H. (2019). Vernakalant and electrical cardioversion for AF—Safe and effective. IJC Heart Vasc..

[103] Hall A.J., Mitchell A. (2019). Introducing Vernakalant into Clinical Practice. Arrhythmia Electrophysiol. Rev..

[104] Antzelevitch C., Burashnikov A. (2009). Atrial-selective sodium channel block as a novel strategy for the management of atrial fibrillation. J. Electrocardiol..

[105] Horváth A., Lemoine M.D., Löser A., Mannhardt I., Flenner F., Uzun A.U., Neuber C., Breckwoldt K., Hansen A., Girdauskas E. (2018). Low resting membrane potential and low inward rectifier potassium currents are not inherent features of hiPSC-derived cardiomyocytes. Stem Cell Rep..

[106] Ravens U., Christ T. (2010). Atrial-selective drugs for treatment of atrial fibrillation. Herzschrittmachertherapie Elektrophysiologie.

[107] Burashnikov A., Belardinelli L., Antzelevitch C. (2012). Atrial-selective sodium channel block strategy to suppress atrial fibril-lation: Ranolazine versus propafenone. J. Pharmacol. Exp. Ther..

[108] Szél T., Koncz I., Jost N., Baczkó I., Husti Z., Virág L., Bussek A., Wettwer E., Ravens U., Papp J.G. (2011). Class I/B an-tiarrhythmic property of ranolazine, a novel antianginal agent, in dog and human cardiac preparations. Eur. J. Pharmacol..

[109] Flenner F., Friedrich F.W., Ungeheuer N., Christ T., Geertz B., Reischmann S., Wagner S., Stathopoulou K., Söhren K.D., Weinberger F. (2016). Ranolazine antagonizes catecholamine-induced dysfunction in isolated cardiomyocytes, but lacks long-term therapeutic effects in vivo in a mouse model of hypertrophic cardiomyopathy. Cardiovasc. Res..

[110] Sag C.M., Mallwitz A., Wagner S., Hartmann N., Schotola H., Fischer T.H., Ungeheuer N., Herting J., Shah A.M., Maier L.S. (2014). Enhanced late INa induces proarrhythmogenic SR Ca leak in a CaMKII-dependent manner. J. Mol. Cell. Cardiol..

[111] Poulet C., Wettwer E., Grunnet M., Jespersen T., Fabritz L., Matschke K., Knaut M., Ravens U. (2015). Late Sodium Current in Human Atrial Cardiomyocytes from Patients in Sinus Rhythm and Atrial Fibrillation. PLoS ONE.

[112] Wu L., Rajamani S., Li H., January C.T., Shryock J.C., Belardinelli L. (2009). Reduction of repolarization reserve unmasks the proarrhythmic role of endogenous late Na+ current in the heart. Am. J. Physiol. Circ. Physiol..

[113] Ravens U. (2010). Antiarrhythmic therapy in atrial fibrillation. Pharmacol. Ther..

[114] De Ferrari G.M., Maier L.S., Mont L., Schwartz P.J., Simonis G., Leschke M., Gronda E., Boriani G., Darius H., Torán L.G. (2015). Ranolazine in the treatment of atrial fibrillation: Results of the dose-ranging RAFFAELLO (Ranolazine in Atrial Fibrillation Following an ELectricaL CardiOversion) study. Heart Rhythm.

[115] Reiffel J.A., Camm A.J., Belardinelli L., Zeng D., Karwatowska-Prokopczuk E., Olmsted A., Zareba W., Rosero S., Kowey P. (2015). HARMONY Investigators. The HARMONY Trial: Combined ranolazine and dronedarone in the management of paroxysmal atrial fibrillation: Mechanistic and therapeutic synergism. Circ. Arrhythm Electrophysiol..

[116] Dobrev D., Friedrich A., Voigt N., Jost N., Wettwer E., Christ T., Knaut M., Ravens U. (2005). The G Protein–Gated Potassium Current I K,ACh Is Constitutively Active in Patients With Chronic Atrial Fibrillation. Circulation.

[117] Bettoni M., Zimmermann M. (2002). Autonomic Tone Variations before the Onset of Paroxysmal Atrial Fibrillation. Circulation.

[118] Kovoor P., Wickman K., Maguire C.T., Pu W., Gehrmann J., Berul C.I., Clapham D.E. (2001). Evaluation of the role of I(KACh) in atrial fibrillation using a mouse knockout model. J. Am. Coll. Cardiol..

[119] Voigt N., Rozmaritsa N., Trausch A., Zimniak T., Christ T., Wettwer E., Matschke K., Dobrev D., Ravens U. (2009). Inhibition of IK,ACh current may contribute to clinical efficacy of class I and class III antiarrhythmic drugs in patients with atrial fibrillation. Naunyn-Schmiedeberg’s Arch. Pharmacol..

[120] Tanaka H., Hashimoto N. (2007). A Multiple Ion Channel Blocker, NIP-142, for the Treatment of Atrial Fibrillation. Cardiovasc. Drug Rev..

[121] Hashimoto N., Yamashita T., Tsuruzoe N. (2008). Characterization of in vivo and in vitro electrophysiological and anti-arrhythmic effects of a novel IK,ACh blocker, NIP-151: A comparison with an IKr-blocker dofetilide. J. Cardiovasc. Pharmacol..

[122] Makary S., Voigt N., Maguy A., Wakili R., Nishida K., Harada M., Dobrev D., Nattel S. (2011). Differential Protein Kinase C Isoform Regulation and Increased Constitutive Activity of Acetylcholine-Regulated Potassium Channels in Atrial Remodeling. Circ. Res..

[123] Kohajda Z., Kristóf A., Kovács P.P., Virág L., Varró A., Jost N. (2010). The properties of the transient outward and ultra-rapid delayed rectifier potassium currents in canine atrial myocytes (FCVB 2010 Meeting-2010, abstract). Cardiovasc. Res..

[124] Juhász V., Hornyik T., Benák A., Nagy N., Husti Z., Pap R., Sághy L., Virág L., Varró A., Baczkó I. (2018). Comparison of the effects of IK,ACh, IKr, and INa block in conscious dogs with atrial fibrillation and on action potentials in remodeled atrial trabeculae. Can. J. Physiol. Pharmacol..

[125] Walfridsson H., Anfinsen O.-G., Berggren A., Frison L., Jensen S., Linhardt G., Nordkam A.-C., Sundqvist M., Carlsson L. (2015). Is the acetylcholine-regulated inwardly rectifying potassium current a viable antiarrhythmic target? Translational discrepancies of AZD2927 and A7071 in dogs and humans. Europace.

[126] Podd S.J., Freemantle N., Furniss S.S., Sulke N. (2016). First clinical trial of specific IK, ACh blocker shows no reduction in atrial fibrillation burden in patients with paroxysmal atrial fibrillation: Pacemaker assessment of BMS-914392 in patients with par-oxysmal atrial fibrillation. Europace.

[127] Pogwizd S.M. (2003). Clinical potential of sodium-calcium exchanger inhibitors as antiarrhythmic agents. Drugs.

[128] Tóth A., Kiss L., Varró A., Nánási P.P. (2009). Potential therapeutic effects of Na+/Ca2+ exchanger inhibition in cardiac diseases. Curr. Med. Chem..

[129] Kovács P.P., Simon J., Christ T., Wettwer E., Varró A., Ravens U. (2010). NCX current is increased in human chronic atrial fibril-lation: A possible explanation for contractile dysfunction? (FCVB 2010 Meeting-2010, abstract). Cardiovasc. Res..

[130] Elias C.L., Lukas A., Shurraw S., Scott J., Omelchenko A., Gross G.J., Hnatowich M., Hryshko L.V. (2001). Inhibition of Na+/Ca2+ exchange by KB-R7943: Transport mode selectivity and antiarrhythmic consequences. Am. J. Physiol. Circ. Physiol..

[131] Birinyi P., Acsai K., Bányász T., Tóth A., Horváth B., Virág L., Szentandrássy N., Magyar J., Varró A., Fülöp F. (2005). Effects of SEA0400 and KB-R7943 on Na+/Ca2+ exchange current and L-type Ca2+ current in canine ventricular cardiomyo-cytes. Naunyn-Schmiedeberg’s Arch. Pharmacol..

[132] Schotten U., Greiser M., Benke D., Buerkel K., Ehrenteidt B., Stellbrink C., Vazquez-Jimenez J.F., Schoendube F., Hanrath P., Allessie M. (2002). Atrial fibrillation-induced atrial contractile dysfunction: A tachycardiomyopathy of a different sort. Cardiovasc. Res..

[133] Kohajda Z., Farkas-Morvay N., Jost N., Nagy N., Geramipour A., Horváth A., Varga R.S., Hornyik T., Corici C., Acsai K. (2016). The Effect of a Novel Highly Selective Inhibitor of the Sodium/Calcium Exchanger (NCX) on Cardiac Arrhythmias in In Vitro and In Vivo Experiments. PLoS ONE.

[134] Wolfes J., Ellermann C., Broer N., Rath B., Willy K., Leitz P.R., Lange P.S., Eckardt L., Frommeyer G. (2020). Antiarrhythmic Effect of Ranolazine in Combination with Selective NCX-Inhibition in an Experimental Model of Atrial Fibrillation. Pharmaceuticals.

[135] Kanter H.L.E., Saffitz J., Beyer E.C. (1992). Cardiac myocytes express multiple gap junction proteins. Circ. Res..

[136] Davis L.M., Rodefeld M.E., Green K., Beyer E.C., Saffitz J.E. (1995). Gap junction protein phenotypes of the human heart and con-duction system. J. Cardiovasc. Electrophysiol..

[137] Solan J.L., Lampe P.D. (2009). Connexin43 phosphorylation: Structural changes and biological effects. Biochem. J..

[138] De Mazière A.M., Scheuermann D.W. (1990). Structural changes in cardiac gap junctions after hypoxia and reoxygenation: A quantitative freeze-fracture analysis. Cell Tissue Res..

[139] Rhett J.M., Ongstad E.L., Jourdan J., Gourdie R.G. (2012). Cx43 Associates with Nav1.5 in the Cardiomyocyte Perinexus. J. Membr. Biol..

[140] Dhein S., Krusemann K., Schaefer T. (1999). Effects of the gap junction uncoupler palmitoleic acid on the activation and re-polarization wavefronts in isolated rabbit hearts. Br. J. Pharmacol..

[141] Carmeliet E. (1999). Cardiac Ionic Currents and Acute Ischemia: From Channels to Arrhythmias. Physiol. Rev..

[142] Coronel R., Fiolet J.W., Wilms-Schopman J.G., Opthof T., Schaapherder A.F., Janse M.J. (1989). Distribution of extracellular potassium and electrophysiologic changes during two-stage coronary ligation in the isolated, perfused canine heart. Circulation.

[143] Lesh M.D., Pring M., Spear J.F. (1989). Cellular uncoupling can unmask dispersion of action potential duration in ventricular myocardium. A computer modeling study. Circ. Res..

[144] Varró A., Baczkó I. (2011). Cardiac ventricular repolarization reserve: A principle for understanding drug-related proarrhythmic risk. Br. J. Pharmacol..

[145] Zaniboni M., Pollard A.E., Yang L., Spitzer K.W. (2000). Beat-to-beat repolarization variability in ventricular myocytes and its suppression by electrical coupling. Am. J. Physiol. Circ. Physiol..

[146] Spach M.S., Heidlage J.F., Barr R.C., Dolber P.C. (2004). Cell size and communication: Role in structural and electrical development and remodeling of the heart. Heart Rhythm.

[147] Wit A.L., Peters N.S. (2012). The role of gap junctions in the arrhythmias of ischemia and infarction. Heart Rhythm.

[148] Kléber A.G., Rudy Y. (2004). Basic Mechanisms of Cardiac Impulse Propagation and Associated Arrhythmias. Physiol. Rev..

[149] Polontchouk L., Haefliger J.-A., Ebelt B., Schaefer T., Stuhlmann D., Mehlhorn U., Kuhn-Regnier F., De Vivie E., Dhein S. (2001). Effects of chronic atrial fibrillation on gap junction distribution in human and rat atria. J. Am. Coll. Cardiol..

[150] Wetzel U., Boldt A., Lauschke J., Weigl J., Schirdewahn P., Dorszewski A., Doll N., Hindricks G., Dhein S., Kottkamp H. (2005). Expression of connexins 40 and 43 in human left atrium in atrial fibrillation of different aetiologies. Heart.

[151] Gutstein D.E., Morley G.E., Vaidya D., Liu F., Chen F.L., Stuhlmann H., Fishman G. (2001). Heterogeneous Expression of Gap Junction Channels in the Heart Leads to Conduction Defects and Ventricular Dysfunction. Circulation.

[152] Papp R., Gönczi M., Kovács M., Seprényi G., Végh Á. (2007). Gap junctional uncoupling plays a trigger role in the antiarrhythmic effect of ischaemic preconditioning. Cardiovasc. Res..

[153] Dhein S., Hagen A., Jozwiak J., Dietze A., Garbade J., Barten M., Kostelka M., Mohr F.-W. (2009). Improving cardiac gap junction communication as a new antiarrhythmic mechanism: The action of antiarrhythmic peptides. Naunyn-Schmiedeberg’s Arch. Pharmacol..

[154] Haugan K., Miyamoto T., Takeishi Y., Kubota I., Nakayama J., Shimojo H., Hirose M. (2006). Rotigaptide (ZP123) improves atrial conduction slowing in chronic volume overload-induced dilated atria. Basic Clin. Pharmacol. Toxicol..

[155] Hennan J.K., Swillo R.E., Morgan G.A., Keith J.C., Schaub R.G., Smith R.P., Feldman H.S., Haugan K., Kantrowitz J., Wang P.J. (2006). Rotigaptide (ZP123) prevents spontaneous ventricular arrhythmias and reduced infarct size during myocardial ischemia/reperfusion injury in open-chest dogs. J. Pharmacol. Exp. Ther..

[156] Igarashi T., Finet J.E., Takeuchi A., Fujino Y., Strom M., Greener I.D., Rosenbaum D.S., Donahue J.K. (2012). Connexin Gene Transfer Preserves Conduction Velocity and Prevents Atrial Fibrillation. Circulation.

[157] Shiroshita-Takeshita A., Sakabe M., Haugan K., Hennan J.K., Nattel S. (2007). Model-dependent effects of the gap junction con-duction-enhancing antiarrhythmic peptide rotigaptide (ZP123) on experimental atrial fibrillation in dogs. Circulation.

[158] Guerra J.M., Everett TH 4th Lee K.W., Wilson E., Olgin J.E. (2006). Effects of the gap junction modifier rotigaptide (ZP123) on atrial conduction and vulnerability to atrial fibrillation. Circulation.

[159] Laurent G., Leong-Poi H., Mangat I., Moe G.W., Hu X., So P.P.-S., Tarulli E., Ramadeen A., Rossman E.I., Hennan J.K. (2009). Effects of Chronic Gap Junction Conduction–Enhancing Antiarrhythmic Peptide GAP-134 Administration on Experimental Atrial Fibrillation in Dogs. Circ. Arrhythmia Electrophysiol..

[160] Aonuma S., Kohama Y., Akai K., Komiyama Y., Nakajima S., Wakabayashi M., Makino T. (1980). Studies on heart. XIX. Isolation of an atrial peptide that improves the rhythmicity of cultured myocardial cell clusters. Chem. Pharm. Bull..

[161] Grover R., Dhein S. (2001). Structure-activity relationships of novel peptides related to the antiarrhythmic peptide AAP10 which reduce the dispersion of epicardial action potential duration. Peptides.

[162] Kjolbye A.L., Knudsen C.B., Jepsen T., Larsen B.D., Petersen J.S. (2003). Pharmacological characterization of the new stable anti-arrhythmic peptide analog Ac-D-Tyr-D-Pro-D-Hyp-Gly-D-Ala-Gly-NH2 (ZP123): In vivo and in vitro studies. J. Pharmacol. Exp. Ther..

[163] Butera J.A., Larsen B.D., Hennan J.K., Kerns E., Di L., Alimardanov A., Swillo R.E., Morgan G.A., Liu K., Wang Q. (2009). Discovery of (2S,4R)-1-(2-Aminoacetyl)-4-benzamidopyrrolidine-2-carboxylic Acid Hydrochloride (GAP-134)13, an Orally Active Small Molecule Gap-Junction Modifier for the Treatment of Atrial Fibrillation. J. Med. Chem..

[164] Müller A., Gottwald M., Tudyka T., Linke W., Klaus W., Dhein S. (1997). Increase in gap junction conductance by an antiarrhythmic peptide. Eur. J. Pharmacol..

[165] Hagen A., Dietze A., Dhein S. (2009). Human cardiac gap-junction coupling: Effects of antiarrhythmic peptide AAP10. Cardiovasc. Res..

[166] Weng S., Lauven M., Schaefer T., Polontchouk L., Grover R., Dhein S. (2002). Pharmacological modification of gap junction coupling by an antiarrhythmic peptide via protein kinase C activation. FASEB J..

[167] Easton J.A., Petersen J.S., Martin P.E. (2009). The anti-arrhythmic peptide AAP10 remodels Cx43 and Cx40 expression and function. Naunyn-Schmiedeberg’s Arch. Pharmacol..

[168] Ueda N., Yamamoto M., Honjo H., Kodama I., Kamiya K. (2014). The role of gap junctions in stretch-induced atrial fibrillation. Cardiovasc. Res..

[169] Hsieh Y.C., Lin J.C., Hung C.Y., Li C.H., Lin S.F., Yeh H.I., Huang J.L., Lo C.P., Haugan K., Larsen B.D. (2016). Gap junction modifier rotigaptide decreases the susceptibility to ventricular arrhythmia by enhancing conduction velocity and suppressing discordant alternans during therapeutic hypothermia in isolated rabbit hearts. Heart Rhythm.

[170] Kjølbye A.L., Haugan K., Hennan J.K., Petersen J.S. (2007). Pharmacological Modulation of Gap Junction Function with the Novel Compound Rotigaptide: A Promising New Principle for Prevention of Arrhythmias. Basic Clin. Pharmacol. Toxicol..

[171] Beardslee M.A., Lerner D.L., Tadros P.N., Laing J.G., Beyer E.C., Yamada K.A., Kléber A.G., Schuessler R.B., Saffitz J.E. (2000). Dephosphorylation and Intracellular Redistribution of Ventricular Connexin43 During Electrical Uncoupling Induced by Ischemia. Circ. Res..

[172] Stahlhut M., Petersen J.S., Hennan J.K., Ramirez M.T. (2006). The Antiarrhythmic Peptide Rotigaptide (ZP123) Increases Connexin 43 Protein Expression in Neonatal Rat Ventricular Cardiomyocytes. Cell Commun. Adhes..

[173] Clarke T.C., Thomas D., Petersen J.S., Evans W.H., Martin P.E.M. (2006). The antiarrhythmic peptide rotigaptide (ZP123) increases gap junction intercellular communication in cardiac myocytes and HeLa cells expressing connexin 43. Br. J. Pharmacol..

[174] Zhao H.-P., Zhang X.-S., Xiang B.-R. (2011). Discontinued drugs in 2010: Cardiovascular drugs. Expert Opin. Investig. Drugs.

[175] Rossman E.I., Liu K., Morgan G.A., Swillo R.E., Krueger J.A., Gardell S.J., Butera J., Gruver M., Kantrowitz J., Feldman H.S. (2009). The gap junction modifier, GAP-134 [(2S,4R)-1-(2-aminoacetyl)-4-benzamido-pyrrolidine-2-carboxylic acid], improves conduction and reduces atrial fibrilla-tion/flutter in the canine sterile pericarditis model. J. Pharmacol. Exp. Ther..

[176] Engstrøm T., Nepper-Christensen L., Helqvist S., Kløvgaard L., Holmvang L., Jørgensen E., Pedersen F., Saunamaki K., Tilsted H.-H., Steensberg A. (2018). Danegaptide for primary percutaneous coronary intervention in acute myocardial infarction patients: A phase 2 randomised clinical trial. Heart.

[177] Ding Y., Prasain K., Nguyen T.D., Hua D.H., Nguyen T.A. (2012). The effect of the PQ1 anti-breast cancer agent on normal tissues. Anti-Cancer Drugs.

[178] Bigelow K., Nguyen T.A. (2014). Increase of gap junction activities in SW480 human colorectal cancer cells. BMC Cancer.

[179] Ding Y., Nguyen T.A. (2013). PQ1, a quinoline derivative, induces apoptosis in T47D breast cancer cells through activation of caspase-8 and caspase-9. Apoptosis.

[180] Chang C.-J., Cheng C.-C., Chen Y.-C., Kao Y.-H., Chen S.-A., Chen Y.-J. (2016). Gap junction modifiers regulate electrical activities of the sinoatrial node and pulmonary vein: Therapeutic implications in atrial arrhythmogenesis. Int. J. Cardiol..

[181] Chen Y.-C., Lu Y.-Y., Cheng C.-C., Lin Y.-K., Chen S.-A., Chen Y.-J. (2014). Sinoatrial node electrical activity modulates pulmonary vein arrhythmogenesis. Int. J. Cardiol..

[182] Schmidt C., Wiedmann F., Schweizer P.A., Katus H.A., Thomas D. (2012). Cardiac two-pore-domain potassium channels (K2P): Physiology, pharmacology, and therapeutic potential. Dtsch. Med. Wochenschr..

[183] Skibsbye L., Poulet C., Diness J.G., Bentzen B.H., Yuan L., Kappert U., Matschke K., Wettwer E., Ravens U., Grunnet M. (2014). Small-conductance calcium-activated potassisium (SK) channels contribute to action potential repolarization in human atria. Cardiovasc. Res..

[184] Yu T., Deng C., Wu R., Guo H., Zheng S., Yu X., Shan Z., Kuang S., Lin Q. (2012). Decreased expression of small-conductance Ca2+-activated K+ channels SK1 and SK2 in human chronic atrial fibrillation. Life Sci..

[185] Zhang Y., Wu H.-J., Che H., Sun H.-Y., Cheng L.-C., Li X., Au W.-K., Tse H.F., Li G.-R. (2013). Functional transient receptor potential canonical type 1 channels in human atrial myocytes. Pflügers Archiv.

[186] Ninio D.M., Saint D.A. (2008). The role of stretch-activated channels in atrial fibrillation and the impact of intracellular acidosis. Prog. Biophys. Mol. Biol..

[187] Heidbüchel H. (2003). A paradigm shift in treatment for atrial fibrillation: From electrical to structural therapy?. Eur. Heart J..

[188] Goette A., Bukowska A., Lendeckel U. (2007). Non-ion channel blockers as anti-arrhythmic drugs (reversal of structural remod-eling). Curr. Opin. Pharmacol..

[189] Dąbrowski R., Szwed H. (2012). Antiarrhythmic potential of aldosterone antagonists in atrial fibrillation. Cardiol. J..

[190] Zhou X., Du J.-L., Yuan J., Chen Y.-Q. (2013). Statins Therapy Can Reduce the Risk of Atrial Fibrillation in Patients with Acute Coronary Syndrome: A Meta-Analysis. Int. J. Med Sci..

[191] Berkowitsch A., Neumann T., Kuniss M., Janin S., Wojcik M., Zaltsberg S., Mitrovic V., Pitschner H.F. (2010). Therapy with Ren-in-Angiotensin system blockers after pulmonary vein isolation in patients with atrial fibrillation: Who is a responder?. Pacing Clin. Electrophysiol..

[192] Veronese G., Montomoli J., Schmidt M., Horváth-Puhó E., Sørensen H.T. (2015). Statin use and risk of atrial fibrillation or flutter: A population-based case-control study. Am. J. Ther..

[193] Fauchier L., Clementy N., Babuty D. (2013). Statin therapy and atrial fibrillation: Systematic review and updated meta-analysis of published randomized controlled trials. Curr. Opin. Cardiol..

[194] Fang W.T., Li H.J., Zhang H., Jiang S. (2012). The role of statin therapy in the prevention of atrial fibrillation: A meta-analysis of randomized controlled trials. Br. J. Clin. Pharmacol..

[195] Alegret J.M., Aragonès G., Elosua R., Beltrán-Debón R., Hernández-Aguilera A., Romero-Menor C., Camps J., Joven J. (2013). The relevance of the association between inflammation and atrial fibrillation. Eur. J. Clin. Investig..

[196] Youn J.-Y., Zhang J., Zhang Y., Chen H., Liu D., Ping P., Weiss J.N., Cai H. (2013). Oxidative stress in atrial fibrillation: An emerging role of NADPH oxidase. J. Mol. Cell. Cardiol..

